# Density Control over MBD2 Receptor-Coated Surfaces Provides Superselective Binding of Hypermethylated DNA

**DOI:** 10.1021/acsami.2c09641

**Published:** 2022-09-02

**Authors:** Ruben W. Kolkman, Sandra Michel-Souzy, Dorothee Wasserberg, Loes I. Segerink, Jurriaan Huskens

**Affiliations:** † Molecular Nanofabrication Group, Department for Molecules & Materials, MESA+ Institute, Faculty of Science and Technology, University of Twente, P.O. Box 217, 7500 AE Enschede, The Netherlands; ‡ BIOS Lab on a Chip Group, MESA+ Institute and TechMed Centre, Max Planck Institute for Complex Fluid Dynamics, Faculty of Electrical Engineering, Mathematics and Computer Science, University of Twente, P.O. Box 217, 7500 AE Enschede, The Netherlands; § Biomolecular Nanotechnology Group, Department for Molecules & Materials, MESA+ Institute, Faculty of Science and Technology, University of Twente, P.O. Box 217, 7500 AE Enschede, The Netherlands

**Keywords:** hypermethylated DNA, MBD2 protein, surface receptor density, self-assembled monolayer, multivalency, superselectivity

## Abstract

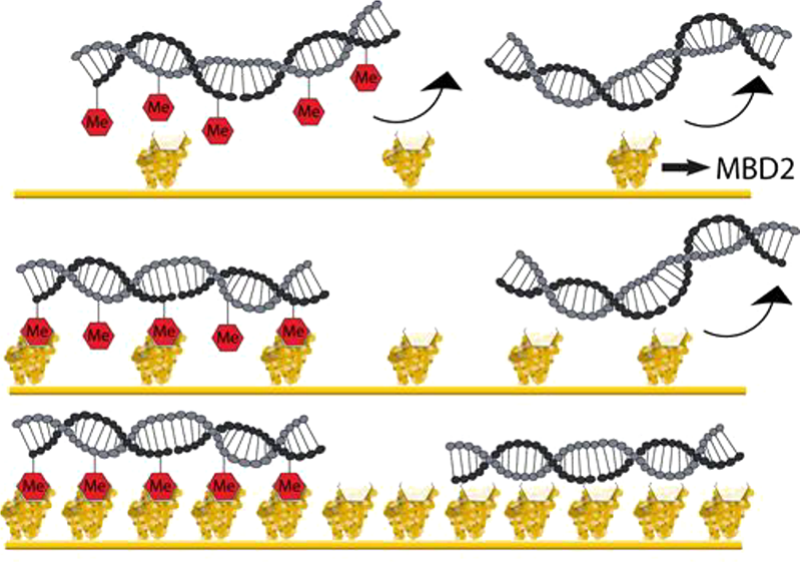

Using the biomarker hypermethylated DNA (hmDNA) for cancer detection requires a pretreatment to isolate or concentrate hmDNA from nonmethylated DNA. Affinity chromatography using a methyl binding domain-2 (MBD2) protein can be used, but the relatively low enrichment selectivity of MBD2 limits its clinical applicability. Here, we developed a superselective, multivalent, MBD2-coated platform to improve the selectivity of hmDNA enrichment. The multivalent platform employs control over the MBD2 surface receptor density, which is shown to strongly affect the binding of DNA with varying degrees of methylation, improving both the selectivity and the affinity of DNAs with higher numbers of methylation sites. Histidine-10-tagged MBD2 was immobilized on gold surfaces with receptor density control by tuning the amount of nickel nitrilotriacetic acid (NiNTA)-functionalized thiols in a thiol-based self-assembled monolayer. The required MBD2 surface receptor densities for DNA surface binding decreases for DNA with higher degrees of methylation. Both higher degrees of superselectivity and surface coverages were observed upon DNA binding at increasing methylation levels. Adopting the findings of this study into hmDNA enrichment of clinical samples has the potential to become more selective and sensitive than current MBD2-based methods and, therefore, to improve cancer diagnostics.

## Introduction

Cancer is accountable for 9.9 million deaths worldwide in 2020, and it is thus one of the deadliest diseases in the world.^1^ To decrease the number of cancer-related deaths, early detection of the disease is essential, since this improves the success rate of treatment.^2,3^ Nationwide screening programs facilitate nowadays the presymptomatic detection of, for example, colorectal cancer^4^ and cervical cancer^5^ by determination of blood in stool and the presence of human papillomavirus in a cervical scrape, respectively. A positive outcome in the colorectal screening requires in general a colonoscopy for disease confirmation. Therefore, both presymptomatic detection methods are invasive and remain uncomfortable for the patient. As an alternative, cancer can also be detected by the analysis of liquid biopsies for the presence of cancer-specific biomarkers.^6,7^ Liquid biopsies are predominantly noninvasive, can handle tumor heterogeneity, and can be used to monitor progression of the disease.^8−10^ However, the identification of suitable biomarkers and sufficiently sensitive detection to measure them in liquid biopsies remains a challenge.

One of the cancer biomarkers found in liquid biopsy samples is hypermethylated DNA (hmDNA).^11,12^ hmDNA is circulating tumor DNA that is epigenetically changed by methylation.^11,12^ The methylation results in the covalent addition of a methyl group to a cytosine base that is followed by a guanine base in the 5′–3′ direction, the so-called CpG region.^13^ Methylation of CpGs occurs to regulate gene transcription, which also takes place in healthy cells.^14^ However, methylation of specific genes, such as tumor suppressor genes, can cause different types of cancer including lung and bladder cancer.^13,15−17^

The usage of hmDNA as a biomarker for cancer detection is currently limited because it is difficult to distinguish hmDNA from nonmethylated DNA.^18−23^ Current methods include the use of methylation-sensitive restriction enzymes,^24−26^ bisulfite conversion,^27,28^ and affinity purification.^29−31^ However, the methods can result in DNA degradation upon bisulfite conversion, incomplete digestion when using a restriction enzyme digest, and selectivity issues during affinity purification.^18,19^ The advantage of affinity purification is that it only involves a molecular recognition binding step followed by an elution step, thus requiring short assay times. Affinity purification of hmDNA is generally used to enrich a solution with hmDNA using beads of which the surfaces are modified with the methyl binding domain-2 (MBD2) protein.^20,30,32,33^ The MBD2 protein is used as a receptor that interacts with a methylated CpG (C*pG, ligand) with a dissociation constant of 3–66 nM.^34−40^ Furthermore, MBD2 has been demonstrated to have one of the best intrinsic binding selectivities among the proteins present within the MBD protein family,^34−40^ with dissociation constant values ranging between 188 and 6500 nM for nonmethylated CpG (CpG).^34,35^ Despite this intrinsic binding selectivity of MBD2 for C*pG, the coenrichment of nonmethylated DNA upon the isolation of hmDNA remains one of the greatest limitations of MBD-based affinity methods.^18,20,21,41^

Here we propose a strategy to improve the separation selectivity using affinity purification on chip by applying the principles of multivalency and superselectivity.^42−46^ Upon the interaction of DNA with an MBD2-modified surface, multiple MBD2-C(*)pG interaction pairs can be formed between a DNA target and the surface.^47,48^ The formation of multiple interaction pairs results in an increase of the avidity, which is attributed to the multivalency effect.^42,49^ Surface binding of methylated or nonmethylated DNA on an MBD2-modified surface is, therefore, only taking place when the avidity is sufficient for surface binding. As a direct result of the multivalent nature, the binding becomes superselective,^43,50,51^ which indicates a sigmoidal, stronger-than-linear dependence of the avidity with MBD2 surface density^43^ and promises a strongly enhanced selectivity of binding of methylated versus nonmethylated DNA.^50^ Furthermore, a hallmark of superselectivity is the occurrence of a so-called threshold receptor density in the binding profile, i.e., the receptor density at which the coverage of the multivalent binder increases from low to high.^43^

Here, we show the development of a method that employs the superselective binding of methylated DNA to achieve hmDNA enrichment at an MBD2-coated surface. In this work we focus on the binding of (methylated) DNA at MBD2-modified surfaces. The importance of controlling the MBD2 surface receptor density with respect to its binding selectivity toward methylated and nonmethylated DNA is assessed. We employ a mixed thiol self-assembled monolayer (SAM) on a gold surface to achieve this density control. The degree of DNA binding on the MBD2-modified surfaces is assessed using up to 90-base pair (bp)-long DNA sequences, with between 0 and 5 C*pGs per sequence. We study the superselectivity of the binding of DNA as a function of CpG methylation to such MBD2-coated surfaces. Furthermore, we compare the maximum surface coverages of methylated DNA and nonmethylated DNA and at which MBD2 densities these are reached. With the developed platform we will demonstrate how the hmDNA isolation selectivity can be improved by optimization of the MBD2 surface receptor density.

## Results and Discussion

### Concept of Superselective hmDNA Enrichment at a Receptor Density-Controlled Platform

**Figure 1:**
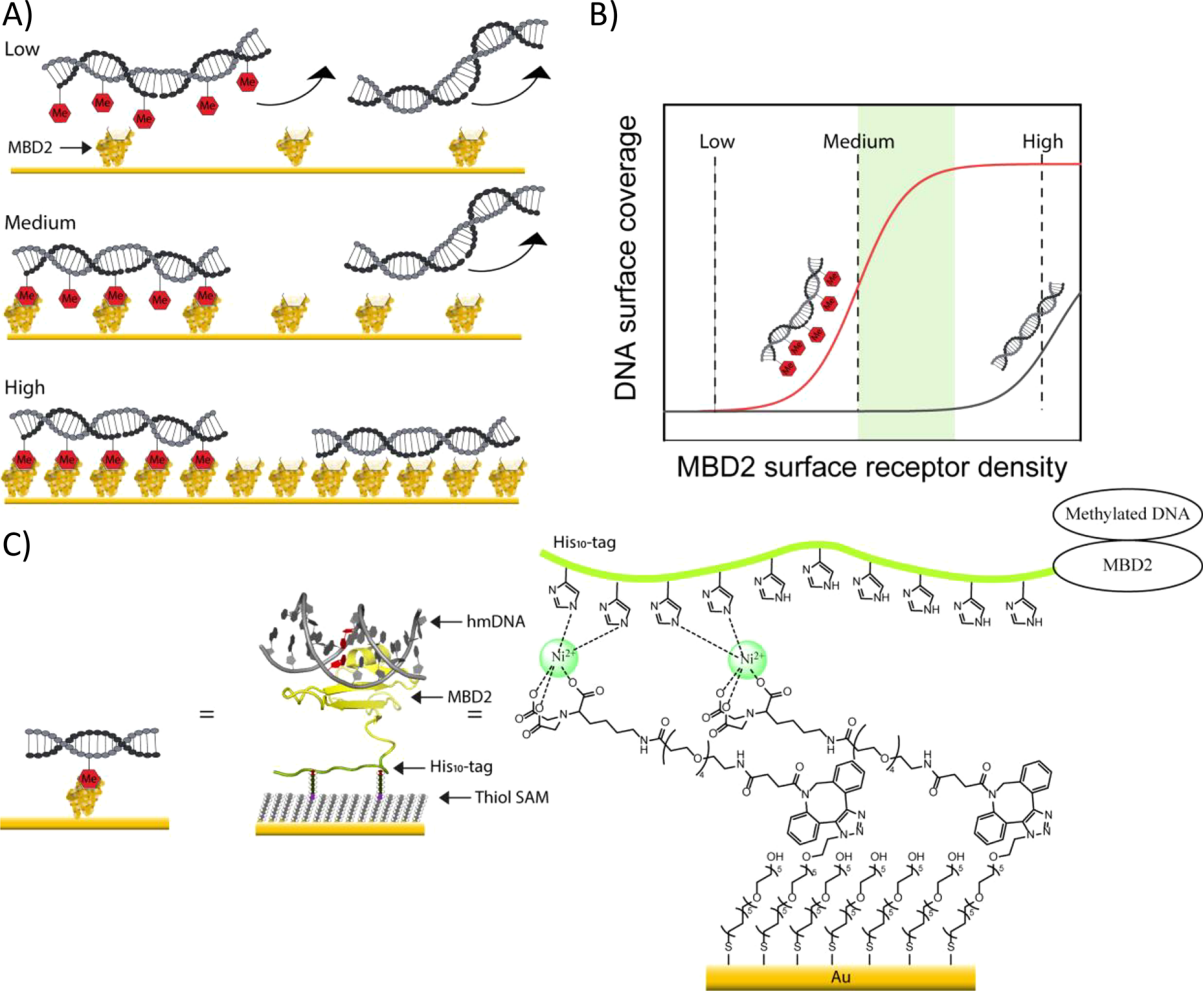
Design of a superselective surface coating for hmDNA enrichment. (A) Schematic illustrations displaying the crucial role of the MBD2 surface receptor density for the superselective binding of methylated DNA at the surface. Low, medium, and high MBD2 surface receptor densities are illustrated and the subsequent degree of interaction with methylated DNA (DNA with red hexagons to represent C*pG) and nonmethylated DNA. (B) The DNA surface coverage on the MBD2-modified surface is displayed as a function of the MBD2 surface receptor density. The optimal MBD2 surface receptor density range to achieve optimal selectivity for enrichment of methylated DNA is indicated in green. (C) Surface chemistry employed to control the degree of His_10_MBD2 immobilization on a gold surface modified with a SAM of functionalized ethylene glycol–alkanethiols. Azide thiols are converted into NiNTA groups which interact with the His_10_ tag of MBD2. Each MBD2 protein interacts with an individual C(*)pG of the DNA. In the middle, the interaction between an immobilized His_10_MBD2 protein (yellow) and a C*pG (red) in a DNA sequence (gray) is displayed, according to the MBD2 crystal structure.^57^ On the right, the mixed ethylene glycol–alkanethiol-based SAM on a gold surface with azide and hydroxyl functional groups is shown. Azide-functionalized thiols are modified with a linker molecule bearing DBCO and NiNTA functional groups. The His_10_ tag of MBD2 is used to achieve immobilization of the protein on the surface.

We aim to develop a superselective hmDNA enrichment platform by tuning the MBD2 surface receptor density (Figure 1). Upon binding of a DNA sequence with a specific number of C(*)pGs at the MBD2-modified surface, the avidity increases at higher degrees of DNA methylation, thereby decreasing the threshold receptor density required for efficient DNA surface binding (Figure 1A). The MBD2 threshold density is defined as the minimal MBD2 density required for significant DNA binding. If the MBD2 density is too low, the possible number of interaction pairs between MBD2 and C(*)pG of DNA that can be formed is limited. In this situation the avidity is insufficient for the surface binding of both types of DNA. Increasing the MBD2 density results in an increase of the number of MBD2-C(*)pG interaction pairs, and as a result, the avidity increases. The avidity increases faster for methylated DNA compared to nonmethylated DNA because of the higher intrinsic affinity of MBD2 for each individual C*pG compared to CpG.^34−40^ When the MBD2 density is increased further, both methylated and nonmethylated DNA can bind strongly. As a direct consequence, we expect there is an optimal density range, at medium MBD2 surface receptor densities at which the MBD2 density is only sufficient for binding methylated DNA, while nonmethylated DNA remains unbound (Figure 1B). In conclusion, control over the MBD2 surface receptor density is crucial for superselective hmDNA enrichment. Additionally, the MBD2 density also controls the total affinity of any bound DNA, and this control is important to fine-tune the efficacy of the surface as a capture layer.

Control over the MBD2 surface receptor density is enabled by employing a mixed SAM on a gold-coated surface of two ethylene glycol–alkanethiols, one with a hydroxyl and the other with an azide end group (Figure 1C). The hydroxyl thiol is the major component and is used to create an antifouling surface,^52−54^ while the minor component, the azide thiol, is used to enable MBD2 immobilization. A linker molecule, bearing a dibenzocyclooctyne (DBCO) moiety on the one end and a nitrilotriacetic acid (NTA) functional group on the other end, was reacted at the azide groups of the SAM by employing the catalyst-free click chemistry reaction between the DBCO and azide functional groups.^55^ The NTA functional groups were subsequently complexed with Ni^2+^ ions to form NiNTA moieties. Finally, the MBD2 protein fused with a histidine-10 (His_10_) tag at the N-terminus (His_10_MBD2) was immobilized at the surface via the NiNTA complexes. An individual NiNTA moiety has the possibility to interact with two histidines with a *K*_d_ of 14 μM.^56^ Therefore, the number of NiNTA moieties interacting with the His_10_ tag is maximally 5. The MBD2 surface receptor density was varied by tuning the stoichiometric ratio between the hydroxyl and azide-functionalized thiols in the SAM, leading to controlled variation of the density of NiNTA groups as well as that of the MBD2s binding to these groups.

### Controlling the MBD2 Surface Receptor Density

**Figure 2:**
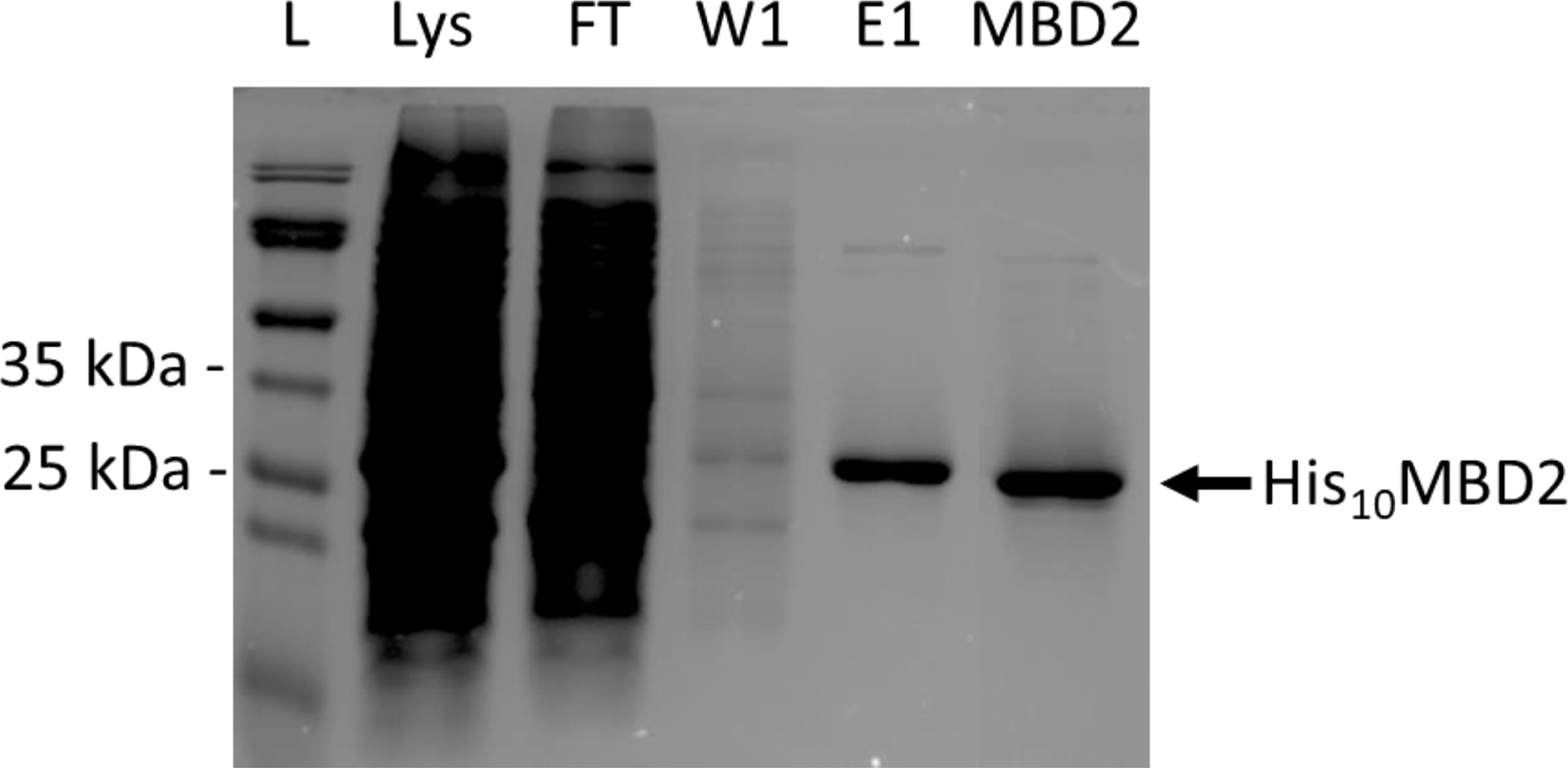
SDS–PAGE of the His_10_MBD2 after isolation from *E. coli* Rosetta DE3 cells using NiNTA column chromatography and SEC. The gel was stained with Coomassie Blue prior to imaging. In the SDS–PAGE gel the ladder (L), cell lysate (Lys), flow through (FT), wash of the NiNTA column (W1), elution of the MBD2 with imidazole (E1), and the isolated His_10_MBD2 after SEC (MBD2) are visible. The characteristic MBD2 band is visible at the expected molecular weight (≈32 kDa).

His_10_MBD2 was produced using *E. coli* Rosetta (DE3) competent cells. The genetic information for the protein was based on the work of Bird et al. (Table S1).^58^ The successful production and purification of His_10_MBD2 were confirmed by sodium dodecyl sulfate–polyacrylamide gel electrophoresis (SDS–PAGE, Figure 2). The protein was isolated from lysed cells using NiNTA affinity column chromatography. The His_10_MBD2 protein was eluted from the NiNTA column by employing an imidazole wash step. Thereafter, the His_10_MBD2 protein was purified by size exclusion chromatography (SEC) and eluted in the immobilization buffer (IB). The final purity of the His_10_MBD2 (molecular weight of ≈32 kDa) was confirmed by the presence of a single strong band at the expected molecular weight upon SDS–PAGE, thereby confirming the purity of the isolated His_10_MBD2 sample.

**Figure 3:**
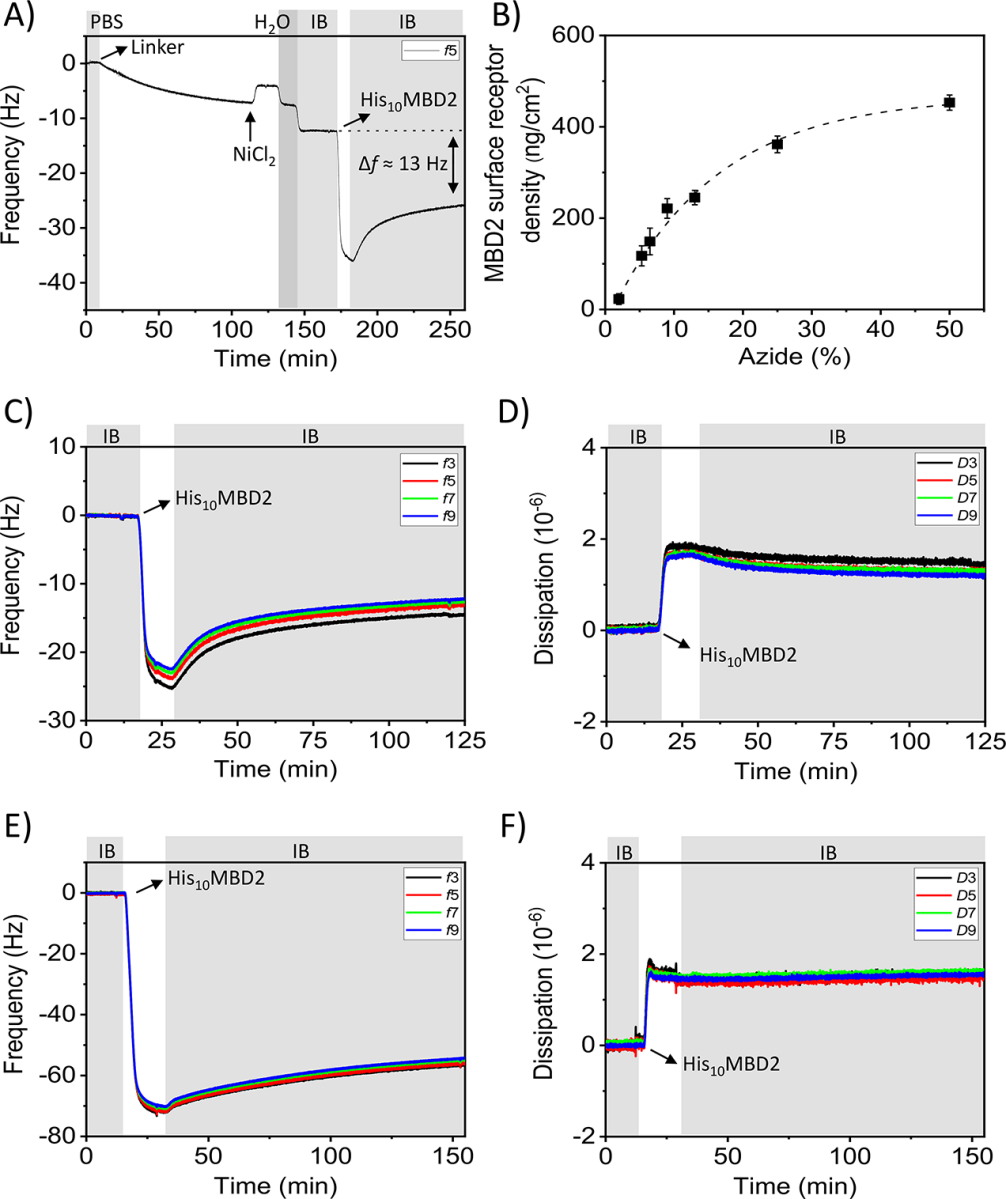
(A) *In situ* monitoring of the His_10_MBD2 immobilization process by QCM. Prior to QCM measurement, a mixed SAM of hydroxyl and azide ethylene glycol–alkanethiols (95:5) was formed overnight. After obtaining a stable baseline, a solution with the DBCO-NTA linker molecule was flown over the SAM. Then, the NTA groups were complexed by NiCl_2_, followed by the immobilization of His_10_MBD2. Washing steps with PBS, Milli-Q (H_2_O), and IB are indicated by the gray areas. (B) MBD2 surface receptor density (determined after the 1.5 h washing step with IB) as a function of the percentage of azide-functionalized thiol in the underlying SAM determined with QCM. Δ*f*_5_ values of irreversibly immobilized MBD2 were converted to MBD2 surface receptor densities using the Sauerbrey equation.^59^ Each data point was obtained from at least two experiments. An exponential trendline was fitted to the data with *R*^2^ > 0.99 to guide the eye. (C, E) Frequency and (D, F) dissipation monitoring over time upon His_10_MBD2 immobilization at overtones 3, 5, 7, and 9 using a SAM with (C, D) 5% azide or (E, F) 50% azide-functionalized thiols in the SAM.

SAMs on gold substrates for quartz crystal microbalance (QCM) analysis were made by overnight immersion in mixtures of azide (minor) and hydroxyl-functionalized (major) ethylene glycol–alkanethiols in varying ratios. After mounting the sample inside a QCM chamber, all subsequent steps to bind MBD2 were monitored *in situ* by QCM (Figure 3). Figure 3A shows a typical example of MBD2 immobilization with 5% azide in the thiol mixture. After obtaining a stable baseline in buffer, a solution with the linker molecule bearing the DBCO and NTA functional groups was flown over the SAM substrate. After 1.5 h a stable baseline was obtained, indicating completion of the reaction. Next, the NTA functional groups were complexed with Ni^2+^ by flushing a solution of NiCl_2_ over the chip, followed by a washing step with water. Thereafter, the His_10_MBD2 protein was immobilized on the surface. Upon the immobilization of MBD2, a typical binding curve was observed, with an initially rapid frequency decrease which leveled off quickly due to surface saturation. The total frequency shift (Δ*f*) of irreversibly immobilized MBD2 is 13 Hz for 5% azide, which has been determined by comparing the baselines in IB prior to and after the addition of MBD2.

The MBD2 surface receptor density is directly related to the percentage of azide in the underlying SAM (Figures 3B and S1–S7). We assume that the measured MBD2 frequency shifts are proportional to the MBD2 surface coverage. The QCM frequency shifts, Δ*f*, of MBD2 at different azide fractions in the SAM then become a mass comparison measure according to the Sauerbrey equation.^59^ This comparison seems valid as the trends for different overtones vary only marginally at both low and high MBD2 surface receptor densities, and the dissipation changes (Δ*D*) were <2 × 10^–6^ (Figure 3C–F). The Δ*f* of MBD2 can thus be used to express the MBD2 surface receptor density. Therefore, the Δ*f* values are here converted to dry mass coverages (in ng/cm^2^), assuming that 55% of the Δ*f* values are due to hydration of the protein at the monolayer surface.^60^ In comparison, a densely packed streptavidin layer typically shows a frequency shift of around 25 Hz.^61^ Even though streptavidin and MBD2 have different molecular weights and their degrees of hydration (which affects QCM frequencies) may differ as well, we regard the here observed MBD2 frequency values ranging from 35 Hz (279 ng/cm^2^) to 55 Hz (438 ng/cm^2^) as indicative of dense packing because the frequency values level off in this range.

When using between 2% and 10% of azide in the SAM, the amount of irreversibly immobilized MBD2 increases linearly as a function of the amount of azide in the SAM. The coverage levels off above 10% azide in the SAM and reaches a maximally packed MBD2 surface density at approximately 40% azide in the SAM. The relationship between the stoichiometric ratio of functional groups in the SAM and the degree of protein immobilization is in agreement with previously reported protein immobilization studies on thiol-based SAM systems.^62,63^ Overall, we conclude that the MBD2 surface receptor density can be tuned by the stoichiometric ratio of hydroxyl and azide-functionalized ethylene glycol–alkanethiols in the SAM.

### Binding Methylated and Nonmethylated DNA to MBD2 Surfaces

**Figure 4:**
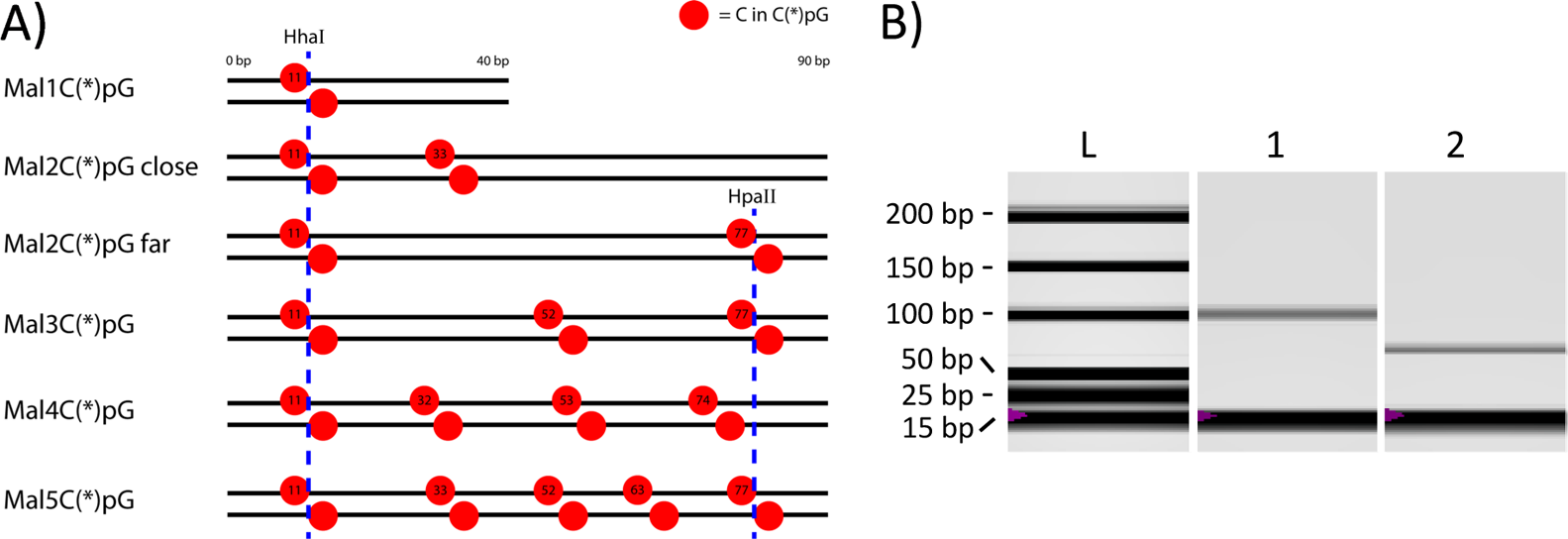
(A) The model DNA targets, based on the Mal gene,^64,65^ are 40 bp or 90 bp in length and consist of 1 (40 bp), 2, 3, 4, or 5 C(*)pGs (90 bp). Location of cytosines in C(*)pG is displayed here by the red circles with nucleotide numbers at the upper strand in the 5′–3′ direction. The methylation-dependent restriction endonucleases HhaI and HpaII recognition sites are indicated by the blue dashed lines. (B) Gel image generated by DNA electrophoresis of Mal3C(*)pG treated with HhaI and HpaII, showing the ladder (L) and HhaI and HpaII-digested Mal3C*pG (1) and Mal3CpG (2). The band at 15 bp in each lane is a marker.

The binding response of DNA with varying degrees of CpG methylation on MBD2-modified surfaces was studied using a model DNA target sequence based on the Mal gene (Figure 4A).^64,65^ In cancer cells, each of the 5 CpGs of the natural Mal gene is methylated.^64,65^ The selected model DNA target is 40 or 90 bp long and contains up to 5 CpGs. To study the effect of different numbers of C(*)pG sites on binding to the MBD2 surfaces, we used the model DNA target with numbers of C(*)pGs ranging between 0 and 5 on the model DNA target. The used DNA sequences in this study are abbreviated as Mal*x*C(*)pG, where *x* represents the number of C(*)pGs in the model DNA target. The nucleotide sequences of the used model DNA targets can be found in Table S2. Varying the number of C(*)pG sites on the model DNA target was used, instead of using very different sequences, to minimize the effect of altering the binding kinetics upon the interaction of different DNA sequences with MBD2, as was reported by Fraga et al.^35^ Mal2C(*)pG was used in two different versions: the C(*)pGs were located either close to or far away from each other with 22 or 66 bp separation between the C(*)pGs, respectively, and these were abbreviated as Mal2C(*)pG-far/close, respectively. For the Mal1C(*)pG, a length of only 40 bp was taken, but we assume this has little effect on the affinity since only monovalent binding is possible in this case. The location of the C(*)pGs in Mal4C(*)pG is slightly shifted compared to the other model DNA targets in order to evaluate the role of the C(*)pG location in a DNA sequence. For example, the second C(*)pG is located at the 32nd and 33rd nucleotide for Mal4C(*)pG and Mal5C(*)pG, respectively.

The CpGs of all Mal*x*CpG constructs were methylated to C*pGs using the *M.SssI* enzyme in the presence of *S*-adenosylmethionine. As a typical example, successful methylation of the Mal3CpG sequence was confirmed by the blockage of digestion upon reacting the methylated sequence with the methylation-dependent restriction endonucleases HhaI and HpaII, as was characterized by DNA electrophoresis (Figure 4A,B). HhaI and HpaII can digest DNA at their recognition sequences, 5′-GCGC-3′ and 5′-CCGG-3′, respectively, in the absence of CpG methylation. Both HhaI and HpaII have one recognition site on Mal3CpG, resulting in the reduction of the sequence length of 24 bp in the case of absence in CpG methylation. Electrophoresis showed retention of the full length for HhaI and HpaII-treated Mal3C*pG, while full digestion into the 66 bp product was observed for Mal3CpG. These results therefore confirm the successful methylation of the former sequence.

**Figure 5:**
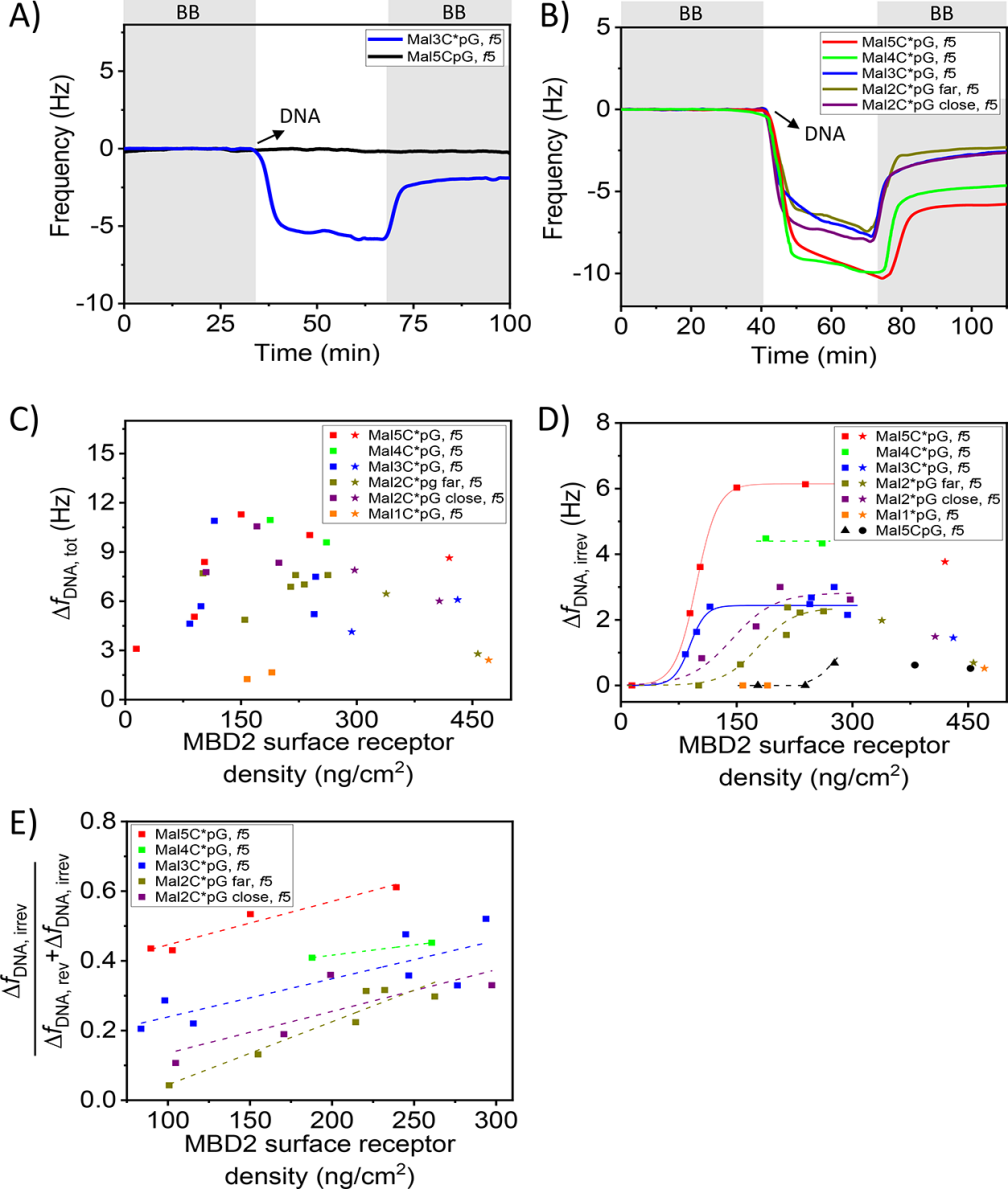
(A) QCM time traces of the binding of 500 nM of Mal5CpG and of Mal3C*pG at MBD2-modified surfaces with receptor densities of 239 ng/cm^2^ and 98 ng/cm^2^, respectively. Washing steps with BB are indicated by the gray areas in the graph. (B) DNA binding profiles of 500 nM Mal5C*pG (red), Mal4C*pG (green), Mal3C*pG (dark blue), and Mal2C*pG far (dark yellow) and close (purple) on MBD2-modified surfaces with a receptor density close to 263 ng/cm^2^. Frequency changes for the (C) total amount (Δ*f*_DNA,tot_) and (D) irreversible fraction (Δ*f*_DNA,irrev_) of model DNA target with varying numbers of C(*)pG sites as a function of the MBD2 surface receptor density measured by QCM. The DNA concentration used was 500 nM for each DNA type. The numbers of C*pGs per sequences are one (orange), two (dark yellow and purple, either far or close), three (dark blue), four (green), and five (red). DNA binding at MBD2 surface receptor densities of >300 ng/cm^2^ and <300 ng/cm^2^ (300 ng/cm^2^ indicates dense protein packing, see text) is indicated by the star and square symbols for the various methylated DNA types, respectively. (D) Mal5CpG lacking CpG methylation is indicated by the black colored triangles (Δ*f*_MBD2_ < 300 ng/cm^2^) and spheres (Δ*f*_MBD2_ > 300 ng/cm^2^). The data points of Mal5C*pG and Mal3C*pG were fitted by a logistic fit; dashed curves provide similar trends as guide to the eye. (E) Irreversible fraction of DNA binding (Δ*f*_DNA,irrev_/(Δ*f*_DNA,irrev_ + Δ*f*_DNA,rev_)) as a function of the MBD2 surface receptor density given that Δ*f*_DNA,rev_ = Δ*f*_DNA,tot_ – Δ*f*_DNA,irrev._ Linear trend lines were fitted through the data points as a guide to the eye.

The binding of the model DNA targets at MBD2-modified surfaces with varying MBD2 surface receptor densities was monitored by QCM (Figure 5). A typical experiment is displayed in Figure 5A for the binding of Mal3C*pG and Mal5CpG at MBD2-coated surfaces with receptor densities corresponding to 98 ng/cm^2^ and 239 ng/cm^2^, respectively (full adsorption processes are shown in Figures S12B and S8B). The negative control Mal5CpG was tested at a higher MBD2 density to promote its binding. Yet, no change in frequency was observed for Mal5CpG at the MBD2 surface, despite the higher receptor density and the higher number of CpG sites. This demonstrates the weak binding of nonmethylated DNA at the MBD2 surface (at least up to MBD2 densities of 239 ng/cm^2^) and confirms the antifouling properties of the MBD2-based capture layer. In contrast, during the first few minutes upon introducing the solution of Mal3C*pG, a sharp decrease of the frequency, signifying binding, was observed, followed by a rapid leveling off of the frequency change. Upon switching back to the binding buffer (BB), a significant part of the bound Mal3C*pG was washed away from the surface. The desorption of DNA can likely be attributed to a fraction of loosely bound DNA, while another fraction remained bound. We tentatively attribute this behavior to two distinct types of DNA binding occurring at the MBD2-modified surface: reversible and irreversible surface binding of DNA. The total amount of bound Mal3C*pG is reflected by the frequency shift after the binding step (Δ*f* = 5.5 Hz), while the irreversibly bound Mal3C*pG is the amount of bound DNA determined after the washing step with BB (Δ*f* = 1.5 Hz). We attribute the firmly bound fraction to DNA binding with a number of C*pG-MBD2 interactions that is large enough to achieve strong binding, while the weaker fraction uses a lower number of binding sites. Different numbers of interaction pairs can occur by local MBD2 density variations and/or a backfilling process. DNA binds first strongly with multiple interactions pairs to the mostly empty surface. Consequently, the number of free MBD2 sites lowers during the binding step, decreasing the possible number of interaction pairs that can be formed with newly arriving DNA that (back)fills the leftover spots on the surface, thus resulting in weaker DNA binding.

The adsorption rate of DNA binding at the MBD2-modified surface was evaluated by comparing the DNA binding curves of Mal5C*pG, Mal4C*pG, Mal3C*pG, Mal2C*pG-close, and Mal2C*pG-far at an MBD2 surface receptor density of ≈263 ng/cm^2^ as obtained from QCM experiments (Figure 5B). During the first minutes of DNA binding, the adsorption rates are comparable for the various types of DNA. This is expected as the mass transport rates of the various DNA types are identical due to the equal DNA lengths (90 bp). After the first minutes of DNA binding, the rate is reduced significantly for all DNA types. Full surface coverage has not been achieved during the 30 min DNA binding step as the DNA binding speed remains constant until switching to buffer. We take 30 min as a relevant time to start washing because we assume the binding sites are largely occupied, and the additionally binding DNA is mostly binding with a lower affinity and will be removed again during washing. Wash steps are continued until a stable plateau in frequency is reached, which signifies the retention of the irreversible DNA fraction.

The total amount of DNA binding as well as that of the irreversible fraction appeared to be dependent on the degree of DNA methylation and on the MBD2 surface receptor density (Figure 5C,D). MBD2 proteins were immobilized on SAM-modified surfaces at varying surface receptor densities followed by quantification of the DNA binding steps (Figures S8–S14). The degree of DNA binding was monitored for all the model DNA targets with 1–5 C*pGs and with 5 CpGs over the entire MBD2 surface receptor density range. The reversible fraction of DNA binding can be deduced by subtracting the irreversible frequency change from the total change; see Figure 5E.

As can be seen in both Figure 5C and Figure 5D, the DNA surface coverage of Mal5C*pG, Mal3C*pG, and Mal2C*pG (close and far) decreases at high MBD2 surface receptor densities (roughly above 300 ng/cm^2^). This decrease in DNA surface coverage is likely caused by steric hindrance between the MBD2 proteins at these high MBD2 densities. Steric hindrance is likely to result in a reduction of the accessible number of active sites and, therefore, results in a decrease in avidity. Consequently, it cannot be concluded from the current data whether the maximum observed DNA surface coverage for Mal2C*pG (close and far), Mal1C*pG, and Mal5CpG is affected by steric hindrance between the MBD2 proteins and, therefore, has reached the upper plateau or not. We therefore restrict most of our analysis to MBD2 densities up to 300 ng/cm^2^.

For the design of a selective DNA capture device, the irreversible fraction is the most relevant and may most accurately represent the fraction that binds with the highest possible valency as explained above. For Mal5C*pG and Mal3C*pG a clear sigmoidal trend was observed (Figure 5D) for the amount of (irreversible) DNA binding as a function of the MBD2 surface receptor density. At low MBD2 densities, the binding of both types of DNA is absent. The lack of binding is attributed to a too low avidity as the number of MBD2-C*pG interaction pairs that can be formed is limited. At increasing MBD2 surface receptor densities a sharp increase in the degree of binding is visible for both sequences. A further increase of the MBD2 surface receptor density results in reaching a maximum surface coverage of Mal5C*pG and Mal3C*pG. The increase of DNA binding from zero to the upper plateau takes place within a narrow range of MBD2 surface receptor densities. This observed DNA binding response is characteristic for superselective systems.^43,50,51^ For both Mal5C*pG and Mal3C*pG, the threshold MBD2 densities correspond to ≈88 ng/cm^2^. Though not tested as extensively, Mal4C*pG follows the same trend. At the threshold density observed for 3 and 5 C*pGs, no strong, irreversible DNA binding of the model DNA targets with two or fewer C*pGs per sequence occurs. All of these sequences require at least an MBD2 coverage of 159 ng/cm^2^ before detectable DNA adsorption occurs. For Mal2C*pG-close and -far, the surface binding response is comparable, indicating minimal effect of the C*pG location on the binding efficiency of a sequence. However, the binding responses of both Mal2C*pG-close and -far do differ notably from those of Mal5C*pG and Mal3C*pG. First, for both Mal2C*pG (close and far), the threshold MBD2 density increases to ≈159 ng/cm^2^. Second, a lower degree of superselectivity (a less steep sigmoidal trend) is observed for both Mal2C*pG in comparison to Mal5C*pG and Mal3C*pG. Both the increase of the threshold MBD2 density and the lower degree of superselectivity are attributed to a reduction of the number of C*pGs per sequence, i.e., the ligand valency, and the concomitantly lower avidities.^43^ Furthermore, notable surface binding of Mal1C*pG and Mal5CpG requires MBD2 surface receptor densities of >239 ng/cm^2^, which is almost a 3-fold increase in the MBD2 surface receptor density compared to the MBD2 threshold for Mal5C*pG and Mal3C*pG. Overall, the MBD2 surface receptor density has been shown to control the superselective binding of methylated DNA and to tune the DNA surface binding according to its number of methylation sites.

Figure 5D also shows different upper plateau levels for DNAs with varying degrees of methylation. The frequencies of the upper plateau levels decrease according to valency from Mal5C*pG to Mal4C*pG to Mal3C*pG. The location of the C*pGs in the Mal4C*pG sequence is different compared to the other types of DNA, but its binding follows the trend set by Mal5C*pG and Mal3C*pG. Therefore, we assume a minimal effect of the C*pG location on the DNA binding at MBD2-modified surfaces. The decreasing plateau trend can likely be attributed to a decrease in the overall affinity due to the decreasing number of interacting sites. On the other hand, the maximum total amounts of bound DNA at varying methylation levels are more comparable to each other below 300 ng/cm^2^ of MBD2 (Figure 5C). We attribute this to the fact that the total bound DNA comprises both the reversible and irreversible DNA fractions and that more than one MBD2-C*pG interaction per DNA are already sufficient for significant transient DNA binding.

Despite the irreversible fraction being most relevant for our targeted application, analyzing trends by comparing the reversibly and irreversibly binding fractions is illustrative for studying the underlying binding mechanism. The ratio between the reversible and irreversible fractions of the total amounts of DNA that bind to the MBD2-modified surface (for MBD2 densities up to 300 ng/cm^2^) is directly related to the degree of methylation of the DNA (Figure 5E). In all cases with two or more C*pGs, the fraction of irreversible binding increases with the MBD2 density. This behavior highlights the dependence on the receptor density, which is a hallmark of multivalent binding. The highest irreversible fractions are observed for Mal5C*pG, followed by Mal4C*pG, Mal3C*pG, and Mal2C*pG-close/far; for 1 C*pG and 5 CpGs, no binding was observed at these MBD2 densities. Thus, up to 61% of the total amount of bound Mal5C*pG binds irreversibly at the MBD2 platform. This trend is again attributed to the valency of the sequences and the resulting differences in avidity. It can be expected that the irreversible DNA fraction increases even to higher values upon the binding of DNA with higher methylation levels.

## Conclusions

In this study we have demonstrated the development of a superselective multivalent platform that binds methylated DNA and employs the MBD2 surface receptor density as a way to control selectivity and affinity. The MBD2 surface receptor density was controlled by employing a thiol-based SAM and tuning the stoichiometric ratio between hydroxyl and azide-functionalized thiols in the SAM. The degree of (non)methylated DNA binding was dependent on the MBD2 surface receptor density and the methylation status of the DNA. DNA containing 3–5 C*pGs per 90 base pairs displayed superselective binding behavior toward MBD2-modified surfaces. The degree of superselectivity was lower for sequences with fewer methylation sites. The degree of irreversible DNA binding to the MBD2 surface increases at higher degrees of DNA methylation and at higher MBD2 densities, showing the strength of the multivalent control over the capture yield of the surface. Therefore, controlling MBD2 surface receptor density is essential for the superselective enrichment of methylated DNA.

Future work could assess the effects of DNA length and of the degree of methylation upon interaction with MBD2-modified surfaces. For example, the main fragment sizes of DNA isolated from blood and urine samples are approximately 160 bp and 90 bp, respectively.^66,67^ Both a higher degree of methylation and a larger DNA fragment size can be expected to result in a higher avidity to an MBD2-coated surface as more C(*)pG-MBD2 interaction pairs can be formed. Likely, this affects the required MBD2 surface receptor density for (non)methylated DNA surface binding. In addition, the effect of different DNA sequences upon hmDNA enrichment should be investigated, as Fraga et al. have shown that different binding kinetics occurs for various DNA sequences in their interaction with MBD2,^35^ thus also affecting the avidity of (non)methylated DNA toward MBD2 surfaces. Next, the developed superselective hmDNA binding platform should be tested with DNA isolated from cancer cell lines and human body fluids, and improvements compared to currently applied methods should be assessed. Finally, the ability to elute surface-bound DNA using a 2 M NaCl solution^68^ should be evaluated to, for instance, sequence hmDNA-enriched DNA samples for cancer diagnostics.

## Methods

### Chemicals

30% Acrylamide/Bis solution 37 0.5:1, gravity flow columns, Experion DNA chips, Experion DNA 1K reagents and supplies as well as 4× Laemmli sample buffer were obtained from BioRad. Coomassie brilliant blue (Coomassie blue), NaCl, β-mercaptoethanol, dimethyl sulfoxide ≥99.7% (DMSO), EDTA, MgCl_2_ ≥98.0% (MgCl_2_), LB broth, lysozyme from chicken egg white (≥90% lysozyme), phenylmethanesulfonyl fluoride (PMSF), ribonuclease A from bovine pancreas (RNase), deoxyribonuclease I from bovine pancreas (DNase), kanamycin sulfate from *Streptomyces kanamyceticus*, nickel(II) chloride hexahydrate (NiCl_2_), *N*_α_,*N*_α_-bis(carboxymethyl)-l-lysine hydrate ≥97.0%, phosphate-buffered saline (PBS) tablets for 10 mM solution, H_2_SO_4_ 95–97%, 0.2 μm membrane filter, and sodium dodecyl sulfate ≥99% (SDS) were purchased from Sigma-Aldrich. Tris(hydroxymethyl)aminomethane (Tris), isopropyl-β-d-thiogalactopyranoside ≥99% (IPTG), H_2_O_2_ 33%, and ethanol absolute were bought from VWR. Imidazole 99% and Triton X-100 were obtained from ACROS Organics. Ni-NTA-agarose beads were purchased from Protino. PD-10 Sephadex desalting columns were obtained from GE Health. DBCO-PEG_4_-NHS ester was purchased from Click Chemistry Tools. All the DNA sequences were bought from Eurofins Genomics. Nuclease-free water, CpG methyltransferase (M.SssI), 1× NEBuffer 2, *S*-adenosylmethionine, Cutsmart buffer, HhaI, HpaII, and high fidelity polymerase Q5 were purchased from New England Biolabs. HSC_11_(EG)_5_-OH and HSC_11_(EG)_5_-N_3_ were bought from Prochimia. QCM gold-coated chips (QS-QSX301) were obtained from Quantum Design GmbH.

### Cloning and Transformation

The used bacterial strains and plasmids are displayed in Tables 1 and 3, and all the required molecular reagents to enable cloning and transformation were obtained from New England Biolabs. The His_10_MBD2 gene was cloned in a pET-15b vector by Eurofins Genomics. The His_10_MBD2 gene (Table S1) was amplified by PCR using the primers displayed in Table 2, and high fidelity polymerase Q5 and cloned into pRSFDuet vector (Novagen) used the SLIC method^69^ between NdeI-KpnI (MCS2) restriction sites as described before. The amplified DNA obtained from PCR was purified using a Macherey Nagel PCR cleanup kit. Then *E. coli* NovaBlue competent cells were transformed with the SLIC product. Plasmids were isolated and purified using the Qiagen miniprep kit. The plasmids were sequenced by Eurofins Genomics to confirm the sequence. Competent cells of *E. coli* Rosetta strain were transformed with the plasmid.

**Table 1:** Bacterial Strains Used in This Study

*E. coli* strain	genotype/characteristics	origin
Rosetta (DE3)	F-ompT hsdSB(rB– mB−) gal dcm (DE3) pRARE (CamR)	Novagen
NovaBlue	endA1 hsdR17 (rK12– mK12+) supE44 thi-1 recA1 gyrA96 relA1 lac F′[proA+B+ lacIqZΔM15::Tn10] (TetR)	Novagen

**Table 2:** Primer Sequence Used To Amplify the His_10_MBD2 Gene by PCR

primer	5′ → 3′	characteristics
His_10_MBD2 fwd	AGAAGGAGATATACATATGCACCATCACCATCATCACCATCATCACCACGATTGTCCTGCGTTG	for NdeI site in pDuet MCS2
His_10_MBD2 rev	ACCAGACTCGAGGGTACCTTAAGCTTCATCACCGCT	for KpnI site in pDuet MCS2

**Table 3:** Plasmid Type Used in This Study

plasmid	genotype/characteristics	origin
pET-15b	ApR, pT7	Novagen
pRSFDuet	Km^R^, 2 MCS, P_T7_, Ori RSF 1030	Novagen
pRSFDuet His_10_MBD2	pRSFDuet carrying MBD2 gene with His_10_-tag at N-terminus cloned into MCS 2	this study

### His_10_MBD2 Production and Purification

The *E. coli* bacteria were grown up to an optical density (OD) at 600 nm of 0.5 at 37 °C in LB medium with 30 μg/mL of kanamycin. The culture was cooled until it reached 17 °C, followed by the expression of the His_10_MBD2 protein after induction with 1 mM IPTG for 15 h at 17 °C while stirring at 210 rpm. The culture was centrifuged (Allegra 25R) at 5000 rpm for 15 min at 4 °C to sediment the bacteria. The bacteria were lysed with sonication in a lysis buffer of 50 mM Tris-HCl pH 7.2, 300 mM NaCl, 30 mM imidazole, 0.1% β-mercaptoethanol, 1 mM EDTA, 20 mM MgCl_2_, 1 mM PMSF, 0.5 mg/mL lysozyme, 20 μg/mL DNase, 20 μg/mL RNase A. Sonication (Fisherbrand 120) was performed on ice twice for 30 s with a waiting step in between of 2 min. The sonicated samples were centrifuged at 3100 rpm for 15 min at 4 °C, and the supernatant was then centrifuged at 40 000 rpm for 60 min at 4 °C (WX Ultra 90, Thermo Scientific). The supernatant was loaded on a NiNTA column and incubated for 30 min while shaking at 4 °C. The column was washed with 25 mL of washing buffer (50 mM Tris pH 7.2, 300 mM NaCl, 30 mM imidazole, 0.1% β-mercaptoethanol). His_10_MBD2 was removed from the NiNTA column with an elution buffer (50 mM Tris pH 7.2, 300 mM NaCl, 650 mM imidazole, 0.1% β-mercaptoethanol). Directly afterward, the His_10_MBD2 sample was purified with a PD10 column and eluted in the IB buffer (50 mM Tris pH 7.2, 300 mM NaCl, 0.1% β-mercaptoethanol). The His_10_MBD2 sample was aliquoted, snap-frozen, and stored at −80 °C until further use.

### SDS–Polyacrylamide Gel Electrophoresis (SDS–PAGE)

Protein fractions were mixed with 4× Laemmli sample buffer +0.1% β-mercaptoethanol at a 1:4 ratio and analyzed on 15% polyacrylamide gels with gel electrophoresis (BioRad) in a running buffer of 25 mM Tris, 192 mM glycine, and 0.1% SDS. The separated proteins were stained using Coomassie brilliant blue solution consisting of 10% acetic acid, 40% ethanol, 50% Milli-Q water, and 2 g/L Coomassie R250. The stained gel was then unstained with a solution of 10% acetic acid, 40% ethanol, and 50% Milli-Q water, followed by imaging using FluorChem M hardware (Proteinsimple).

### DNA Methylation

DNA methylation was performed by mixing 4 μg of DNA with 8 units of *M.SssI* enzyme, *S*-adenosylmethionine at a concentration of 600 μM and 2.5 μL of NEBuffer 2. The reaction volume was increased to 25 μL with nuclease free water. The reaction was performed at 37 °C for 15 h in a T100 thermocycler (BioRad).

### Methyl-Sensitive Restriction Enzyme Digest

10 ng of DNA was mixed with 1 μL of Cutsmart buffer, 5 units of HhaI, and 5 units of HpaII. The total reaction volume was increased to 10 μL using nuclease free water. Digestion was performed for 15 h at 37 °C using a T100 thermocycler (Biorad). Afterward the enzymes were deactivated by a heat treatment of 85 °C for 20 min.

### DNA Electrophoresis

DNA samples were analyzed using DNA Experion chips and Experion DNA 1K Reagents and Supplies according to the instructions of the manufacturer on an automated electrophoresis system (Experion, BioRad).

### Synthesis of Linker Molecule

DBCO-PEG_4_-NHS was dissolved in DMSO at 250 mM, directly aliquoted, and stored at −18 °C until further use. *N*_α_,*N*_α_-Bis(carboxymethyl)-l-lysine hydrate was dissolved in PBS pH 7.4 at 1 mM before the start of the reaction. The dissolved DBCO-OEG_4_-NHS was added to the *N*_α_,*N*_α_-bis(carboxymethyl)-l-lysine hydrate solution at a final concentration of 0.1 mM. The reaction components were stirred overnight at 180 rpm to ensure completion of reaction.

### SAM Formation

Gold-coated QCM chips were cleaned in piranha solution for 10 s followed by immersion of the chips in Milli-Q water. Afterward, the chips were rinsed extensively with ethanol, Milli-Q water, and ethanol, followed by drying using N_2_. The gold chips were then oxidized using UV-ozone (BioForce, Nanosciences) for 30 min. A thiol solution was prepared using HSC_11_(EG)_5_-OH and HSC_11_(EG)_6_-N_3_ at the desired ratio between the two components in ethanol with a total thiol concentration of 2 mM. The oxidized gold chips were completely immersed in the thiol solution overnight to form the SAM. After the SAM formation the QCM chips were rinsed extensively with ethanol, Milli-Q water, and ethanol and dried in a stream of N_2_.

### Quartz Crystal Microbalance (QCM)

Gold-coated QCM chips modified with the SAM were mounted in the QCM analyzer equipped with four individually addressable flow cells (Biolin Scientific). A flow rate of 30 μL/min was set during the experiments with a peristaltic pump (Biolin Scientific). All solutions were filtered with a 0.2 μm filter prior to use. Frequency and dissipation values used in this study are normalized for the used overtone. After each QCM experiment the system was cleaned with a 15 min washing step with 1% SDS solution continued by Milli-Q water for 15 min. Frequency shift of surface immobilized MBD2 is converted to MBD2 surface receptor densities using the Sauerbrey equation while assuming that 55% of the mass is due to water binding and using a Sauerbrey constant of 17.7 ng/(cm^2^·Hz)

### DNA Binding to MBD2 Surfaces

After SAM formation on the gold-coated QCM chip, the linker molecule was flushed over the surface for 1.5 h at a concentration of 0.1 mM in PBS pH 7.4 followed by the addition of 25 mM NiCl_2_ in Milli-Q water for 10 min and a washing step with Milli-Q for 5 min and, subsequently, IB until the slope was constant. The washing step with Milli-Q was used to prevent the reduction of NiCl_2_ by β-mercaptoethanol present in the IB. His_10_MBD2 dissolved in IB at a concentration of 1 μM was added until stabilization of the signal. After MBD2 immobilization, a washing step with IB for 1.5 h was applied followed by flushing with BB until a stable signal was achieved. BB contains 50 mM Tris, 350 mM NaCl, and 0.1% Triton X-100. Then 500 nM DNA dissolved in BB was added for 30 min followed by a washing step with BB. The MBD2 surface receptor density and degree of DNA binding were determined using the normalized frequency of the fifth overtone (*f*_5_).

## Supporting Information Available

His_10_MBD2 sequence, QCM binding experiments for MBD2 immobilization monitoring, DNA sequences, QCM binding experiments for MBD2 immobilization monitoring, and DNA binding (PDF)
