# Supplementary material for: Density Control over MBD2 Receptor-Coated Surfaces Provides Superselective Binding of Hypermethylated DNA
Source: ACS Appl Mater Interfaces. 2022 Sep 2;14(36):40579–89. doi: 10.1021/acsami.2c09641 (PMC9478954; doi:10.1021/acsami.2c09641)
Supplement: Supplementary file 1 — am2c09641_si_001.pdf [file am2c09641_si_001.pdf]

## Supporting information

# Density control over MBD2 receptor-coated surfaces provides superselective binding of hypermethylated DNA

Ruben W. Kolkman,[a,b] Sandra Michel-Souzy,[c] Dorothee Wasserberg,[b] Loes I. Segerink,*[,b] Jurriaan Huskens*[,a]

[a] Molecular Nanofabrication group, Department for Molecules & Materials, MESA+ Institute, Faculty of Science and Technology, University of Twente, P.O. Box 217, 7500 AE Enschede, The Netherlands

[b] BIOS Lab on a Chip group, MESA+ Institute and TechMed Centre, Max Planck Institute for Complex Fluid Dynamics, Faculty of Electrical Engineering, Mathematics and Computer Science, University of Twente, P.O. Box 217, 7500 AE Enschede, The Netherlands

[c] Biomolecular Nanotechnology group, Department for Molecules & Materials, MESA+ Institute, Faculty of Science and Technology, University of Twente, P.O. Box 217, 7500 AE Enschede, The Netherlands

* Corresponding author: l.i.segerink@utwente.nl, j.huskens@utwente.nl

**Table S1. Gene sequence of His$_{10}$MBD2 based on the MBD2 amino acid sequence with UniProtKB/Swiss-Prot ID: Q9UBB5.1.**[1]

| | 5′ - 3′ |
|---|---|
| His$_{10}$MBD2 gene | ATGCACCATCACCATCATCACCATCATCACCACGATTGTCCTGCGTTGCCGCCC<br>GGATGGAAAAAAGAGGAAGTTATTCGTAAATCTGGTCTGAGTGCGGGCAAGT<br>CAGATGTATATTATTTCTCCCCTTCGGGTAAAAAGTTCCGTAGTAAACCTCAAC<br>TCGCGCGCTACCTTGGCAATACAGTGGATCTCAGTTCTTTCGATTTTCGCACTG<br>GAAAGATGATGCCATCAAAGCTGCAAAAAAATAAACAGCGCCTACGCAACGA<br>CCCACTTAACCAAAATAAAGGCAAACCAGATTTAAATACAACCTTACCCATTCG<br>TCAGACTGCGTCTATTTTTAAACAACCGGTCACCAAAGTAACCAATCATCCGA<br>GTAACAAAGTTAAATCCGACCCGCAACGCATGAACGAGCAACCGCGGCAGTT<br>ATTTTGGGAGAAGCGCTTGCAGGGCCTGTCGGCGTCCGATGTCACCGAACAG<br>ATCATTAAGACCATGGAGTTGCCGAAAGGCCTGCAGGGCGTTGGTCCGGGTA<br>GCAACGACGAGACCCTGCTGTCAGCCGTGGCATCCGCGCTGCACACCAGCAG<br>CGCACCGATTACGGGTCAGGTGTCGGCTGCCGTGGAAAAAAACCCGGCCGTT<br>TGGCTGAACACGTCGCAGCCGCTGTGCAAAGCCTTTATCGTCACGGACGAAG<br>ATATCCGAAAACAGGAAGAACGTGTGCAGCAGGTGCGTAAAAAACTGGAAG<br>AAGCTCTGATGGCCGACATACTGAGCAGAGCAGCAGACACGGAAGAAATGG<br>ATATCGAAATGGATAGCGGTGATGAAGCTTAA |

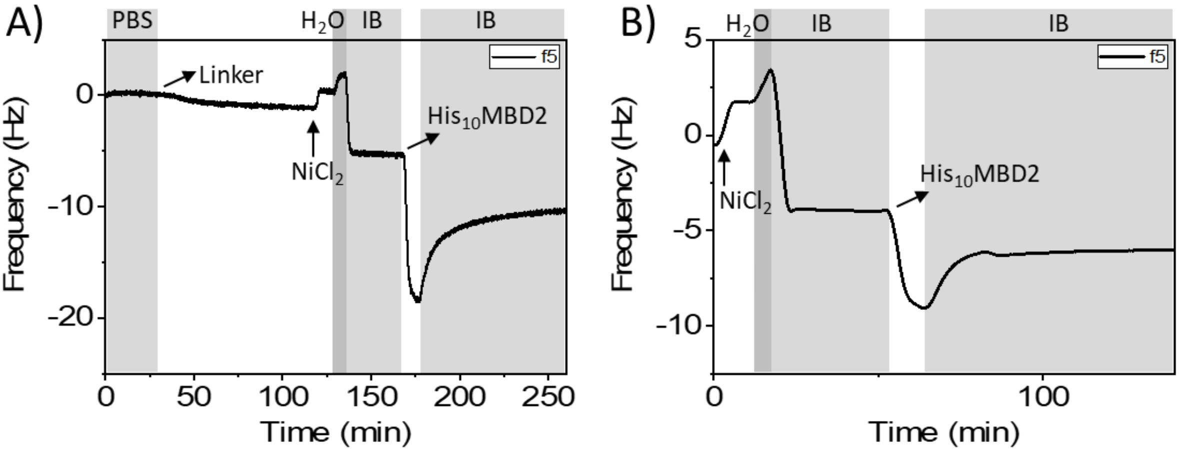


**Figure S1.** *In-situ* monitoring of the His$_{10}$MBD2 immobilization process by QCM. Prior to QCM measurement, a SAM with two ethylene glycol-alkanethiols was formed overnight with 2% azide functionalized thiols. The monitoring with QCM starts with reacting the DBCO-NTA linker molecule onto the SAM. The process is continued by activation of the NTA functional groups using $NiCl_2$ and the immobilization of His$_{10}$MBD2. The MBD2 surface receptor densities at the SAM containing 2% azide was: A) $\Delta f$ = 3.9 Hz and B) $\Delta f$ = 2.8 Hz. Washing steps with PBS, Milli-Q ($H_2O$) and IB are indicated by the grey areas.

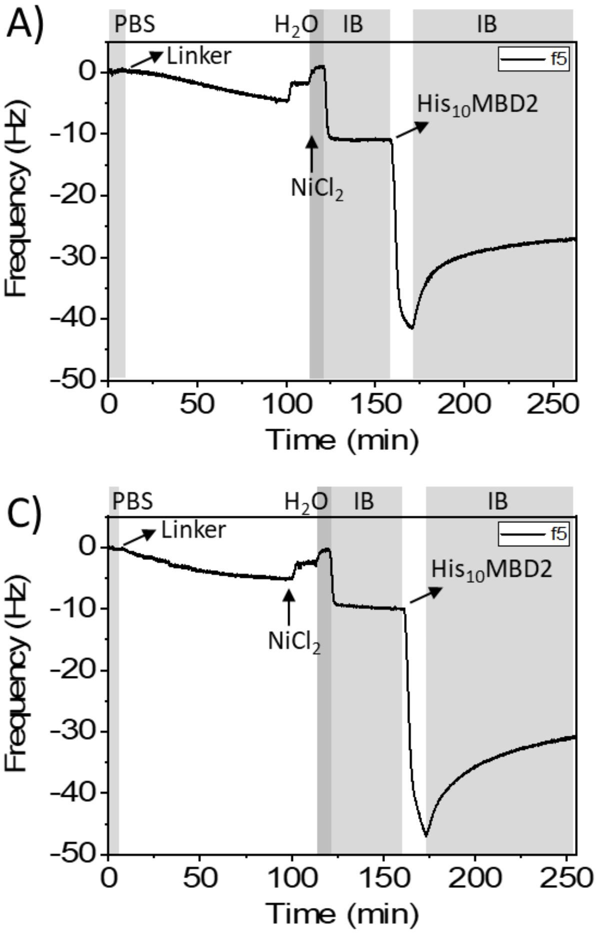


**Figure S2.** *In-situ* monitoring of the His$_{10}$MBD2 immobilization process by QCM. Prior to QCM measurement, a SAM with two ethylene glycol-alkanethiols was formed overnight with 5.3% azide functionalized thiols. The monitoring with QCM starts with reacting the DBCO-NTA linker molecule onto the SAM. The process is continued by activation of the NTA functional groups using $NiCl_2$ and the immobilization of His$_{10}$MBD2. The MBD2 surface receptor densities at the SAM containing 5.3% azide was: A) $\Delta f$ = 16.0 Hz, B) $\Delta f$ = 11.9 Hz, C) $\Delta f$ = 17.99 Hz and D) $\Delta f$ = 13.08 Hz. Washing steps with PBS, Milli-Q ($H_2O$) and IB are indicated by the grey areas.

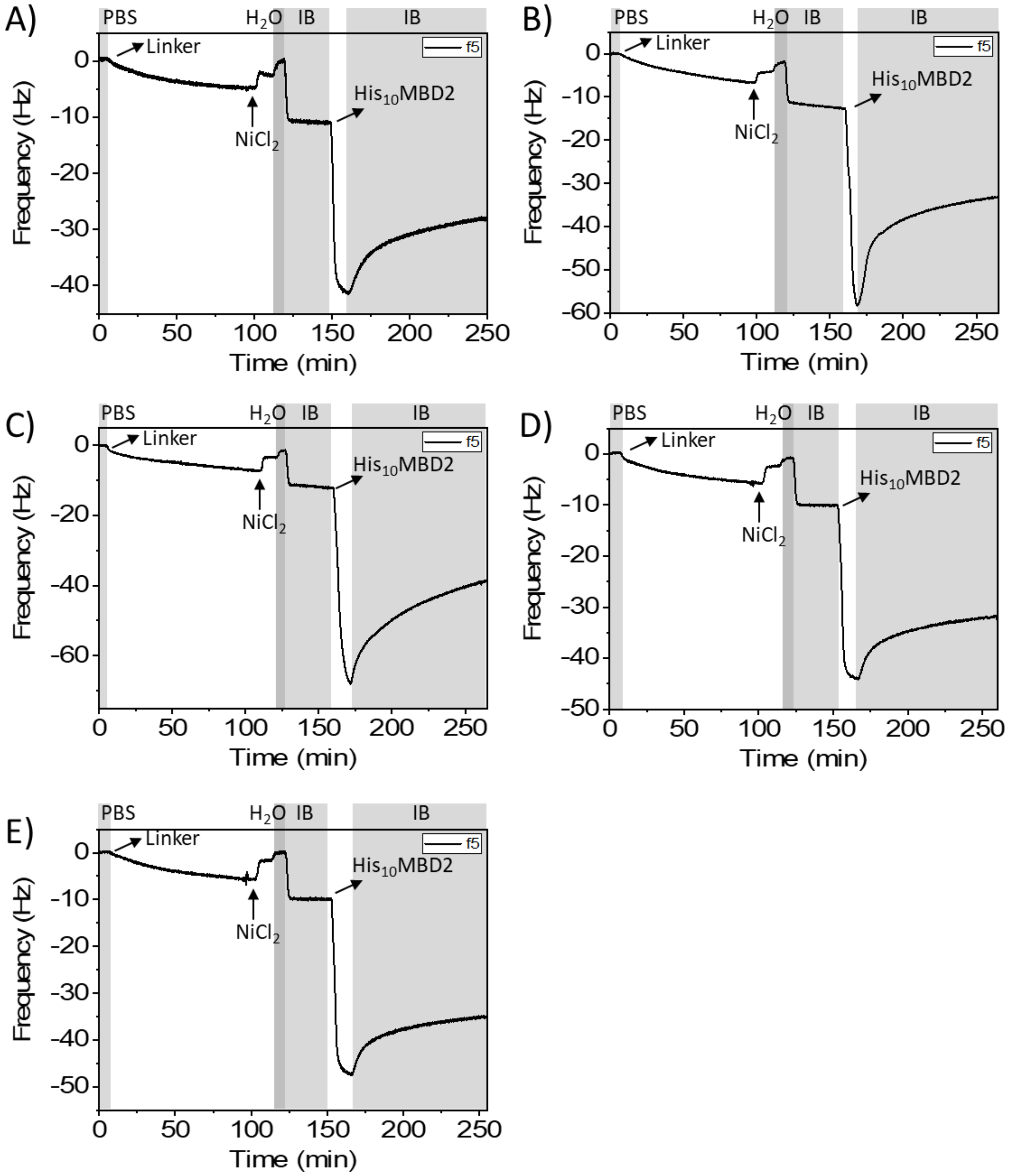


**Figure S3.** *In-situ* monitoring of the His$_{10}$MBD2 immobilization process by QCM. Prior to QCM measurement, a SAM with two ethylene glycol-alkanethiols was formed overnight with 6.5% azide functionalized thiols. The monitoring with QCM starts with reacting the DBCO-NTA linker molecule onto the SAM. The process is continued by activation of the NTA functional groups using $NiCl_2$ and the immobilization of His$_{10}$MBD2. The MBD2 surface receptor densities at the SAM containing 6.5% azide was: A) $\Delta f$ = 14.4 Hz, B) $\Delta f$ = 15.87 Hz, C) $\Delta f$ = 20.5 Hz, D) $\Delta f$ = 19.36 Hz and E) $\Delta f$ = 23.58. Washing steps with PBS, Milli-Q ($H_2O$) and IB are indicated by the grey areas.

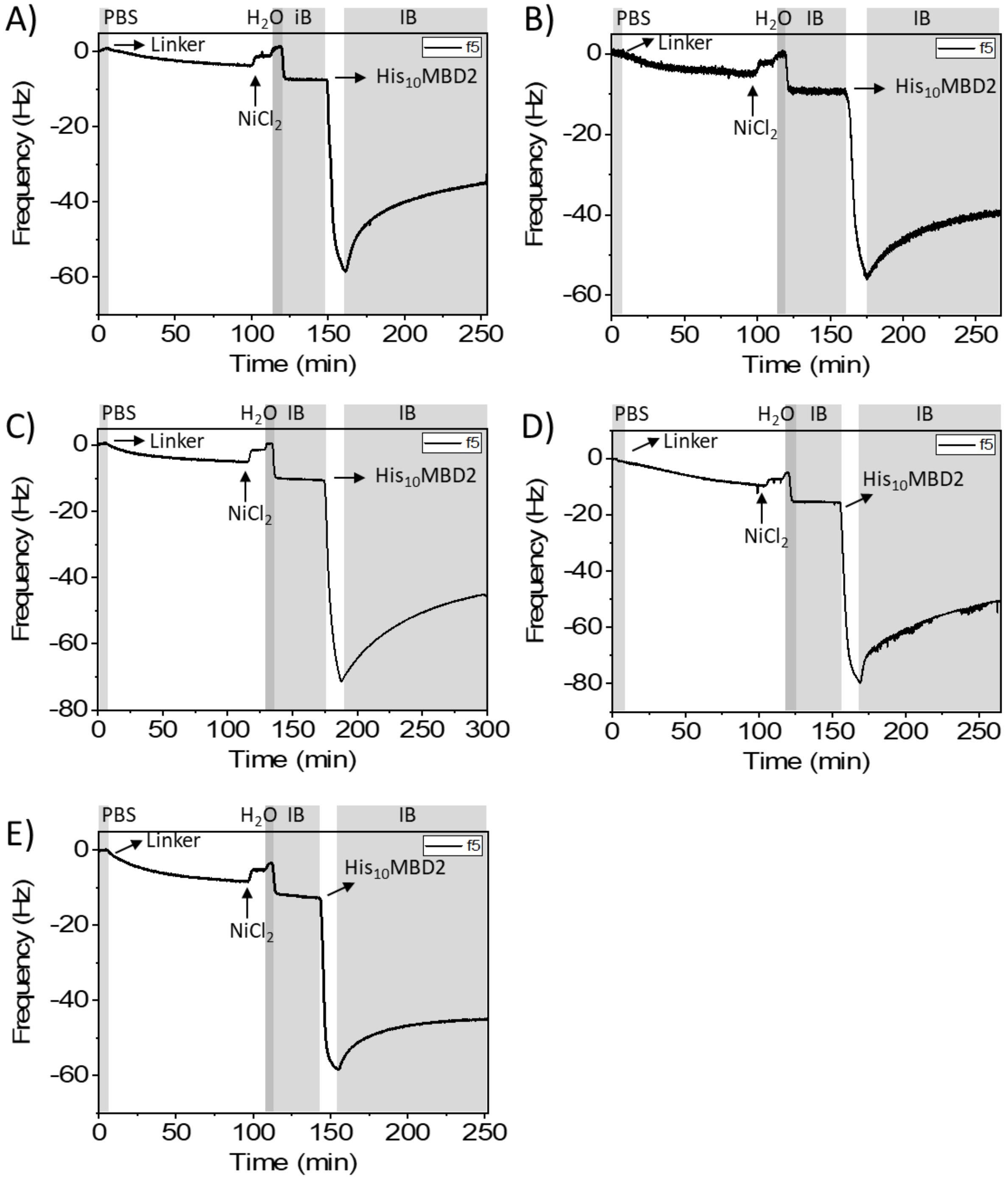


**Figure S4.** *In-situ* monitoring of the His$_{10}$MBD2 immobilization process by QCM. Prior to QCM measurement, a SAM with two ethylene glycol-alkanethiols was formed overnight with 9% azide functionalized thiols. The monitoring with QCM starts with reacting the DBCO-NTA linker molecule onto the SAM. The process is continued by activation of the NTA functional groups using $NiCl_2$ and the immobilization of His$_{10}$MBD2. The MBD2 surface receptor densities at the SAM containing 9% azide was: A) Δ*f* = 23.72 Hz, B) Δ*f* = 27.85 Hz, C) Δ*f* = 30.98 Hz, D) Δ*f* = 29.21 Hz and E) Δ*f* = 27.12. Washing steps with PBS, Milli-Q ($H_2O$) and IB are indicated by the grey areas.

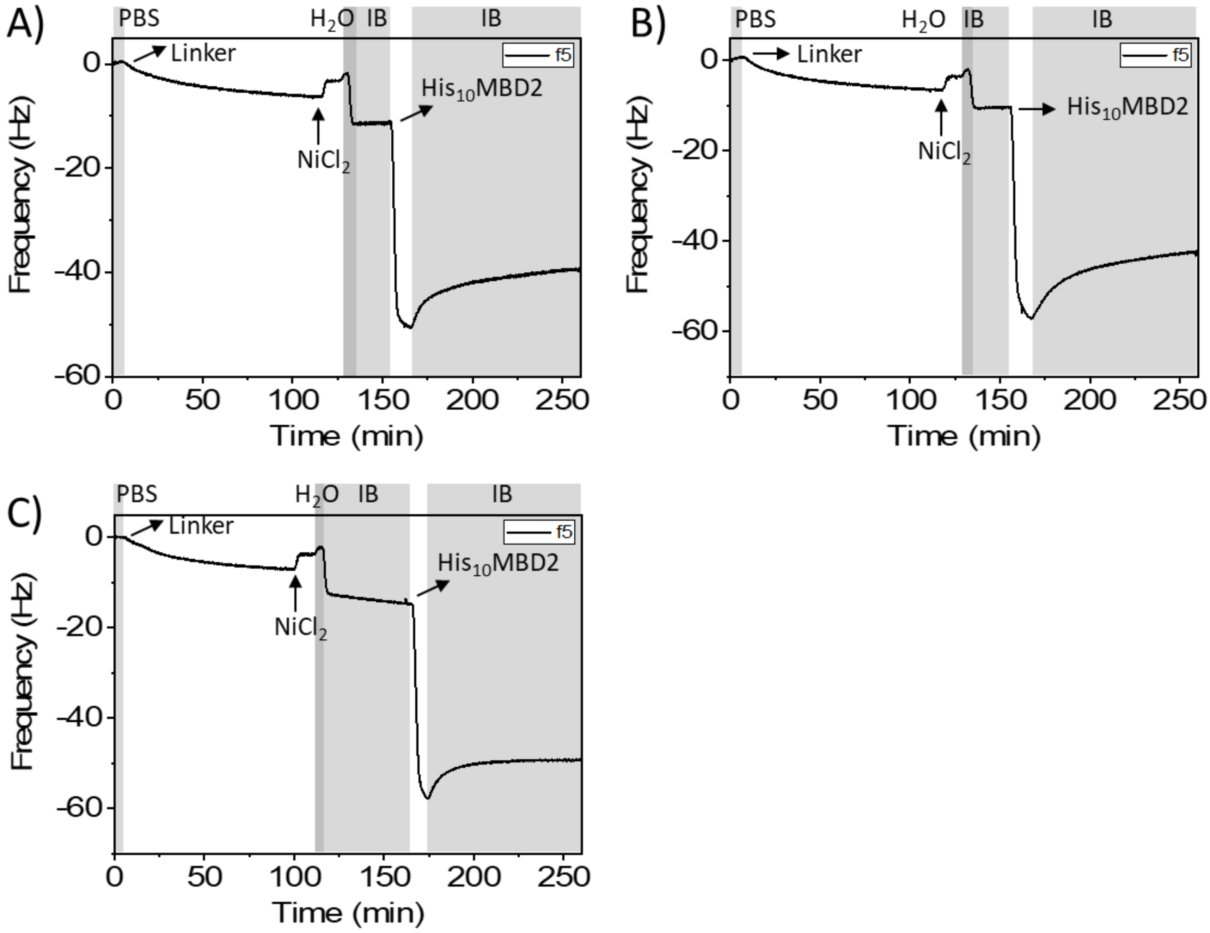


**Figure S5.** *In-situ* monitoring of the $His_{10}$MBD2 immobilization process by QCM. Prior to QCM measurement, a SAM with two ethylene glycol-alkanethiols was formed overnight with 13% azide functionalized thiols. The monitoring with QCM starts with reacting the DBCO-NTA linker molecule onto the SAM. The process is continued by activation of the NTA functional groups using $NiCl_2$ and the immobilization of $His_{10}$MBD2. The MBD2 surface receptor densities at the SAM containing 13% azide was: A) $\Delta f$ = 29.14 Hz, B) $\Delta f$ = 32.97 Hz, C) $\Delta f$ = 30.15 Hz. Washing steps with PBS, Milli-Q ($H_2O$) and IB are indicated by the grey areas.

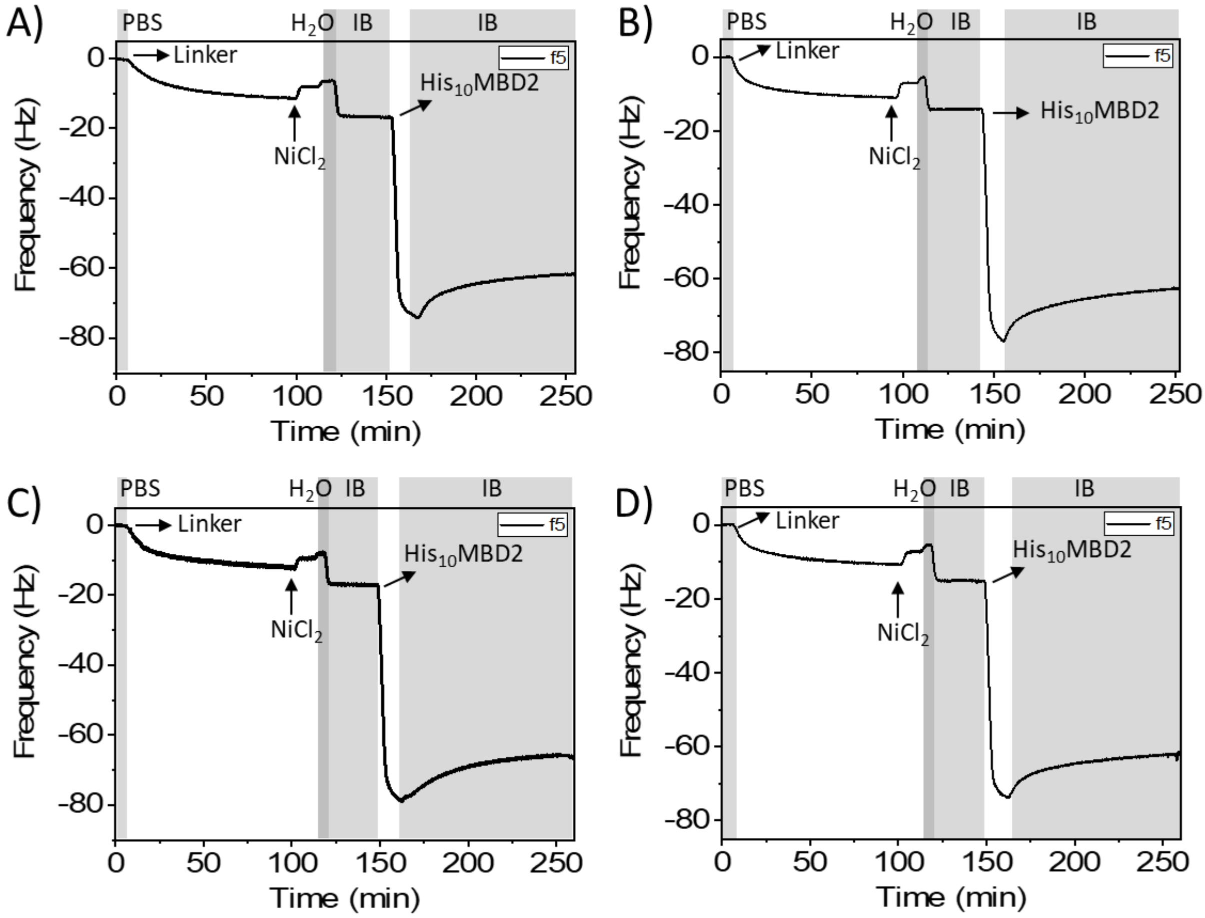


**Figure S6.** *In-situ* monitoring of the $His_{10}$MBD2 immobilization process by QCM. Prior to QCM measurement, a SAM with two ethylene glycol-alkanethiols was formed overnight with 25% azide functionalized thiols. The monitoring with QCM starts with reacting the DBCO-NTA linker molecule onto the SAM. The process is continued by activation of the NTA functional groups using $NiCl_2$ and the immobilization of $His_{10}$MBD2. The MBD2 surface receptor densities at the SAM containing 25% azide was: A) $\Delta f$ = 42.47 Hz, B) $\Delta f$ = 47.83 Hz, C) $\Delta f$ = 46.44 Hz and D) $\Delta f$ = 44.77 Hz. Washing steps with PBS, Milli-Q ($H_2O$) and IB are indicated by the grey areas.

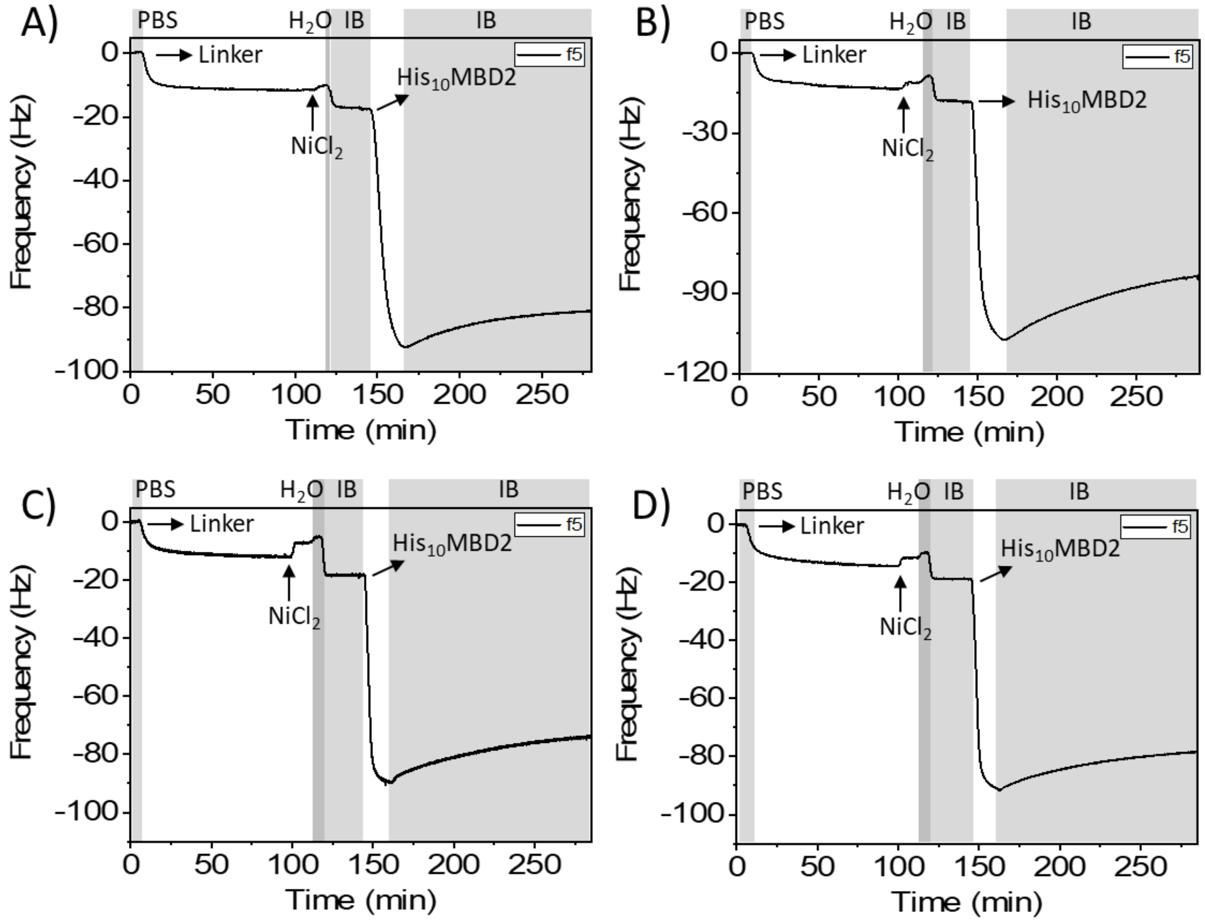


**Figure S7.** *In-situ* monitoring of the $His_{10}MBD2$ immobilization process by QCM. Prior to QCM measurement, a SAM with two ethylene glycol-alkanethiols was formed overnight with 50% azide functionalized thiols. The monitoring with QCM starts with reacting the DBCO-NTA linker molecule onto the SAM. The process is continued by activation of the NTA functional groups using $NiCl_2$ and the immobilization of $His_{10}MBD2$. The MBD2 surface receptor densities at the SAM containing 50% azide was: A) $\Delta f$ = 59.14 Hz, B) $\Delta f$ = 57.36 Hz, C) $\Delta f$ = 54.09 Hz and D) $\Delta f$ = 56.88 Hz. Washing steps with PBS, Milli-Q ($H_2O$) and IB are indicated by the grey areas.

**Table S2. DNA sequences of the used model DNA targets in the 5' – 3'direction. Location of the C(*)pGs are highlighted.**

| | | 5' - 3' |
|---|---|---|
| Mal5C(*)pG | FWD | CAGGCAGATG**CG**CAGCACCAAGCAGAGAGGCC**CG**GTGCAGGATCCCAGGCC**CG**AACCAGGCC**CG**GCTCAGTGGAGC**CG**GAAGGGGCAGGC |
| | REV | GCCTGCCCCTTC**CG**GCTCCACTGAGC**CG**GGCCTGGTT**CG**GGCCTGGGATCCTGCAC**CG**GGCCTCTCTGCTTGGTGCTG**CG**CATCTGCCTG |
| Ma4C(*)pG | FWD | CAGGCAGATG**CG**CAGCACCAAGCAGAGAGGC**CG**GGTGCAGGATCCCAGGCCC**CG**ACCAGGCCAGGCTCAGTGG**CG**CTGGAAGGGGCAGGC |
| | REV | GCCTGCCCCTTCCAG**CG**CCACTGAGCCTGGCCTGGT**CG**GGGCCTGGGATCCTGCACC**CG**GCCTCTCTGCTTGGTGCTG**CG**CATCTGCCTG |
| Mal3C(*)pG | FWD | CAGGCAGATG**CG**CAGCACCAAGCAGAGAGGCCAGGTGCAGGATCCCAGGCC**CG**AACCAGGCCTGGCTCAGTGGAGC**CG**GAAGGGGCAGGC |
| | REV | GCCTGCCCCTTC**CG**GCTCCACTGAGCCAGGCCTGGTT**CG**GGCCTGGGATCCTGCACCTGGCCTCTCTGCTTGGTGCTGCGCATCTGCCTG |
| Mal2C(*)pG far | FWD | CAGGCAGATG**CG**CAGCACCAAGCAGAGAGGCCAGGTGCAGGATCCCAGGCCAGAACCAGGCCTGGCTCAGTGGAGC**CG**GAAGGGGCAGGC |
| | REV | GCCTGCCCCTTC**CG**GCTCCACTGAGCCAGGCCTGGTTCTGGCCTGGGATCCTGCACCTGGCCTCTCTGCTTGGTGCTG**CG**CATCTGCCTG |
| Mal2C(*)pG close | FWD | CAGGCAGATG**CG**CAGCACCAAGCAGAGAGGCC**CG**GTGCAGGATCCCAGGCCAGAACCAGGCCTGGCTCAGTGGAGCAGGAAGGGGCAGGC |
| | REV | GCCTGCCCCTTCCTGCTCCACTGAGCCAGGCCTGGTTCTGGCCTGGGATCCTGCAC**CG**GGCCTCTCTGCTTGGTGCTG**CG**CATCTGCCTG |
| Mal1C(*)pG | FWD | CAGGCAGATG**CG**CAGCACCAAGCAGAGAGGCCAGGTGCAG |
| | REV | CTGCACCTGGCCTCTCTGCTTGGTGCTG**CG**CATCTGCCTG |

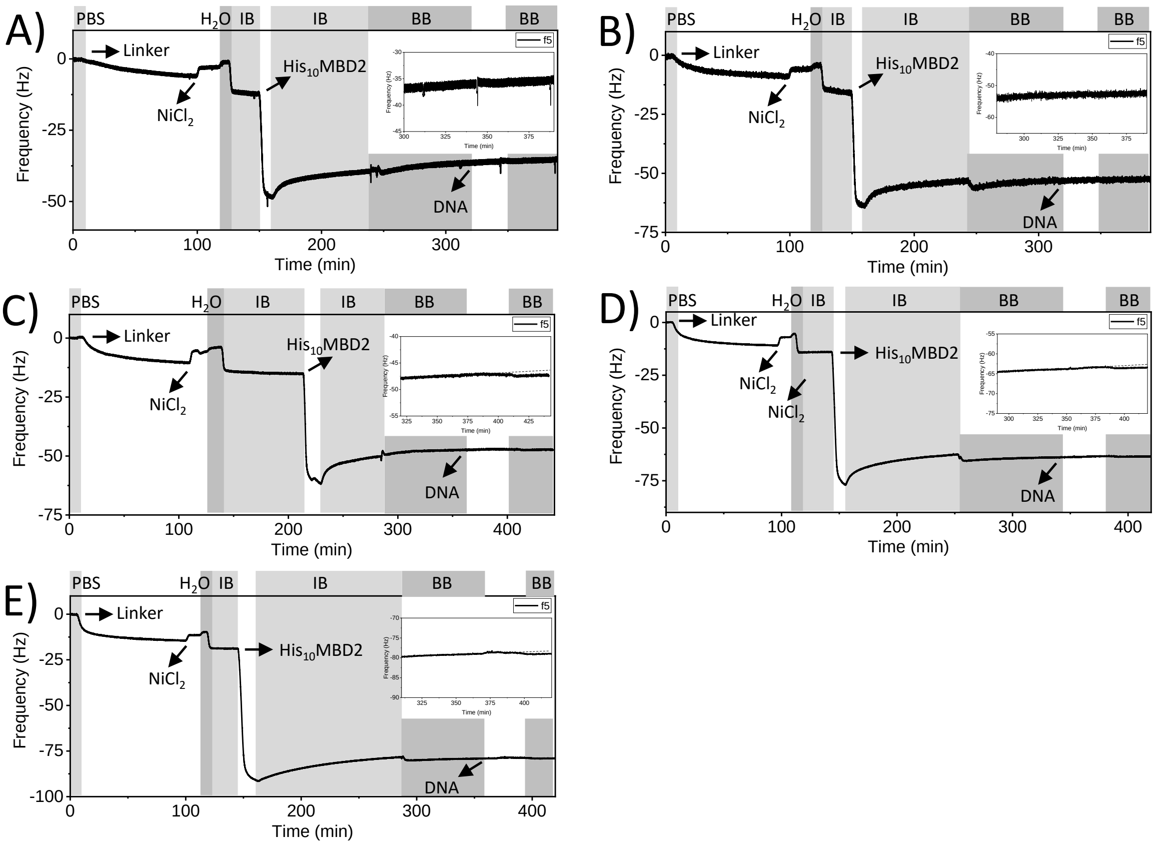


**Figure S8.** QCM time traces of the $His_{10}$MBD2 immobilization and subsequent DNA binding of Mal5CpG. Prior to the $His_{10}$MBD2 immobilization, the SAM was formed, followed by linker binding and activation of the NTA groups by $NiCl_2$. The binding of Mal5CpG was monitored on MBD2 surface receptor densities of: A) $\Delta f$ = 22.3 Hz, B) $\Delta f$ = 30.0 Hz, C) $\Delta f$ = 34.7 Hz, D) $\Delta f$ = 47.8 Hz and E) $\Delta f$ = 56.9. Washing steps with PBS, Milli-Q ($H_2O$), IB and BB are indicated by the grey areas.

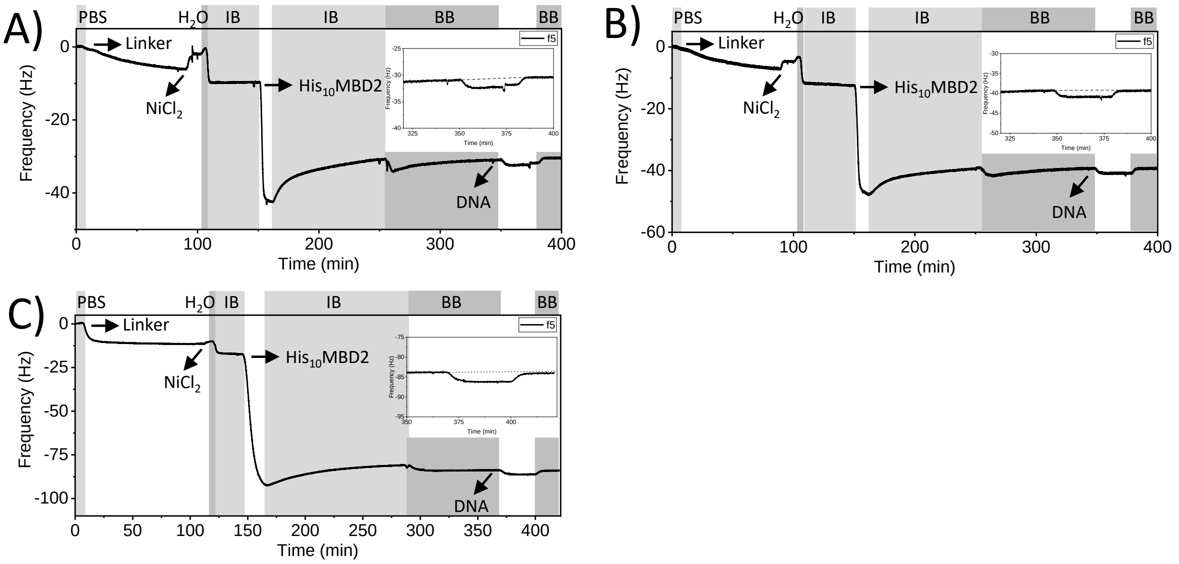


**Figure S9.** QCM time traces of the $His_{10}$MBD2 immobilization and subsequent DNA binding of Mal1C*pG. Prior to the $His_{10}$MBD2 immobilization, the SAM was formed, followed by linker binding and activation of the NTA groups by $NiCl_2$. The binding of Mal1C*pG was monitored on MBD2 surface receptor densities of: A) $\Delta f$ = 19.9 Hz, B) $\Delta f$ = 23.9 Hz and C) $\Delta f$ = 59.1 Hz. Washing steps with PBS, Milli-Q ($H_2O$), IB and BB are indicated by the grey areas.

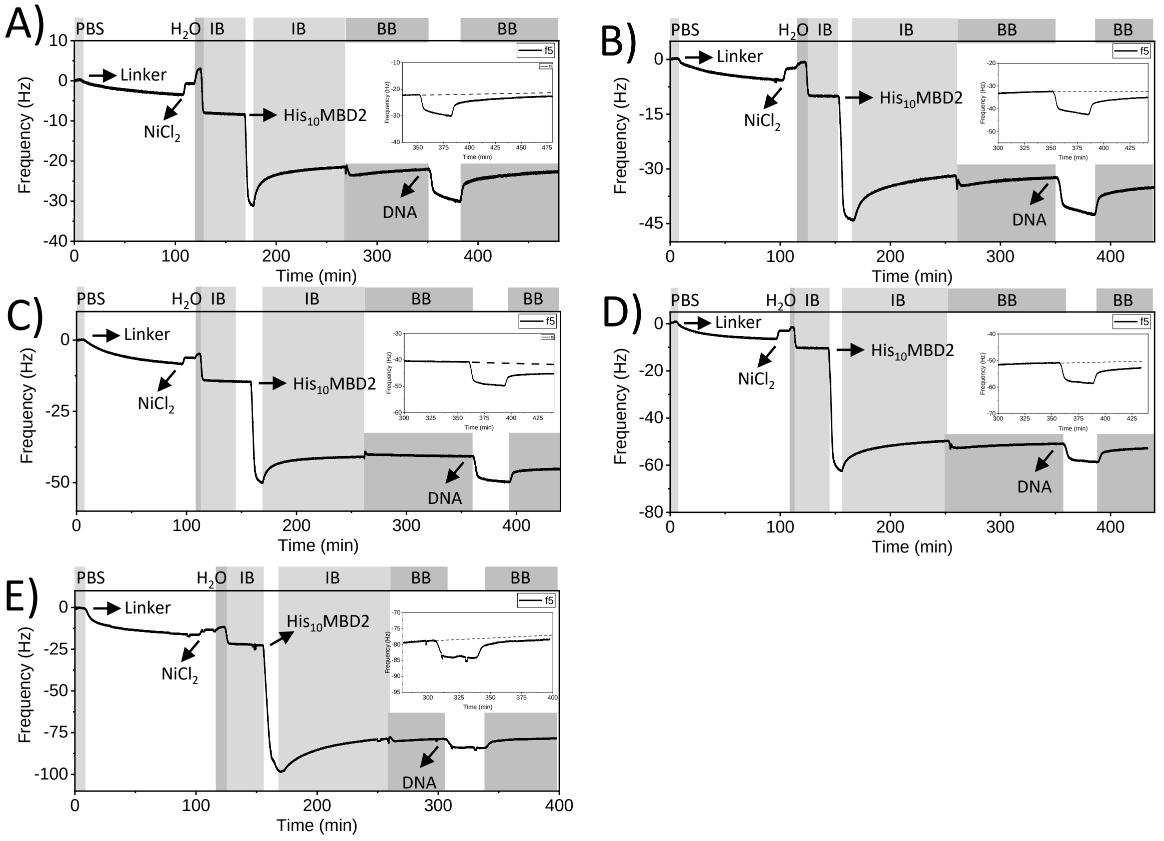


**Figure S10.** QCM time traces of the $His_{10}MBD2$ immobilization and subsequent DNA binding of Mal2C*pG close. Prior to the $His_{10}MBD2$ immobilization, the SAM was formed, followed by linker binding and activation of the NTA groups by $NiCl_2$. The binding of Mal2C*pG close was monitored on MBD2 surface receptor densities of: A) $\Delta f$ = 13.2 Hz B) $\Delta f$ = 22.0 Hz, C) $\Delta f$ = 25.9 Hz, D) $\Delta f$ = 37.3 Hz and E) $\Delta f$ = 51.1 Hz. Washing steps with PBS, Milli-Q ($H_2O$), IB and BB are indicated by the grey areas.

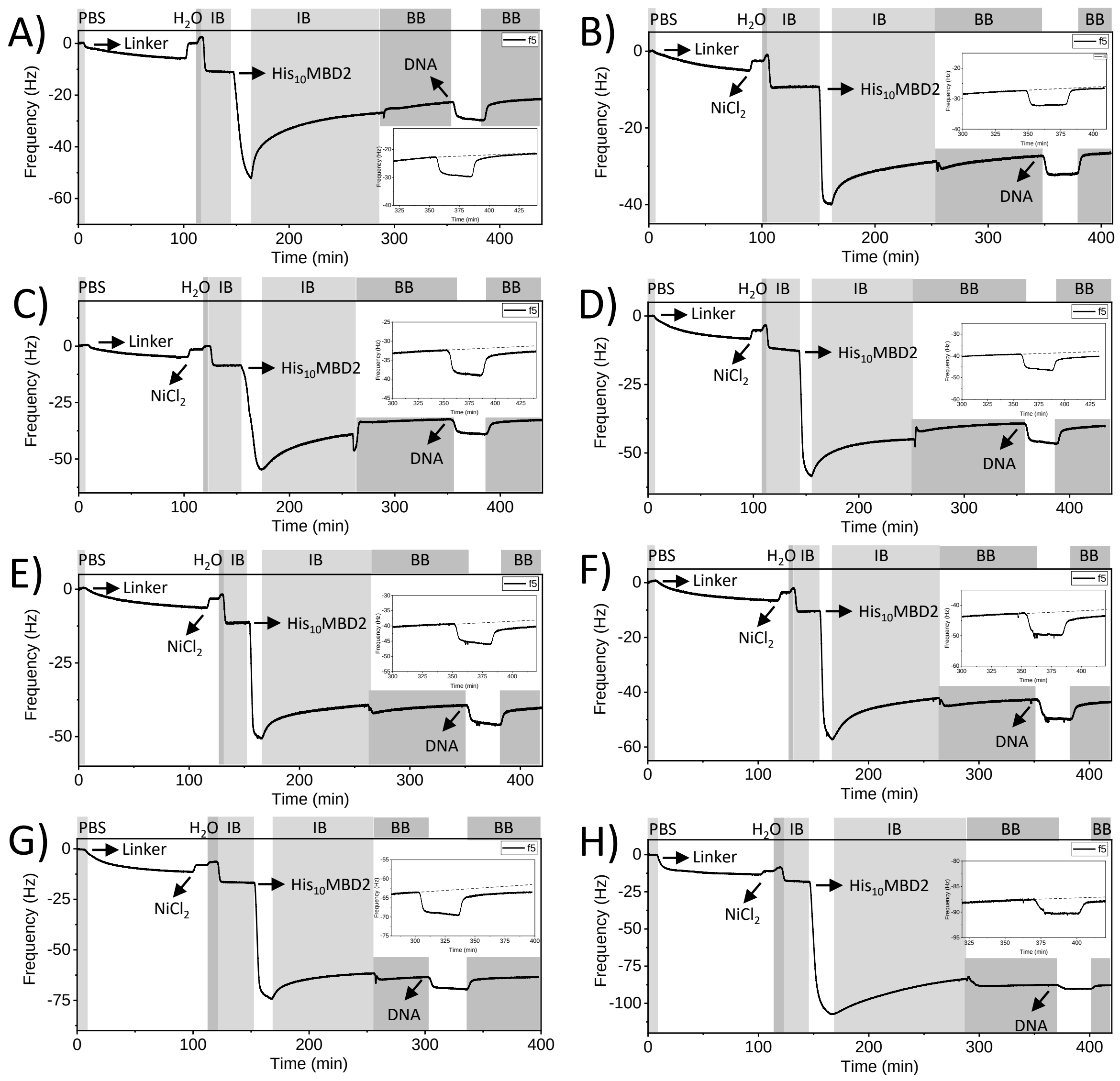


**Figure S11.** QCM times trace of the His$_{10}$MBD2 immobilization and subsequent DNA binding of Mal2C*pG far. Prior to the His$_{10}$MBD2 immobilization, the SAM was formed, followed by linker binding and activation of the NTA groups by NiCl$_2$. The binding of Mal2C*pG far was monitored on MBD2 surface receptor densities of: A) $\Delta f$ = 12.6 Hz, B) $\Delta f$ = 19.5 Hz, C) $\Delta f$ = 26.9 Hz, D) $\Delta f$ = 27.1 Hz, E) $\Delta f$ = 29.1 Hz, F) $\Delta f$ = 33.0 Hz, G) $\Delta f$ = 42.5 Hz and H) $\Delta f$ = 57.4 Hz. Washing steps with PBS, Milli-Q ($H_2O$), IB and BB are indicated by the grey areas.

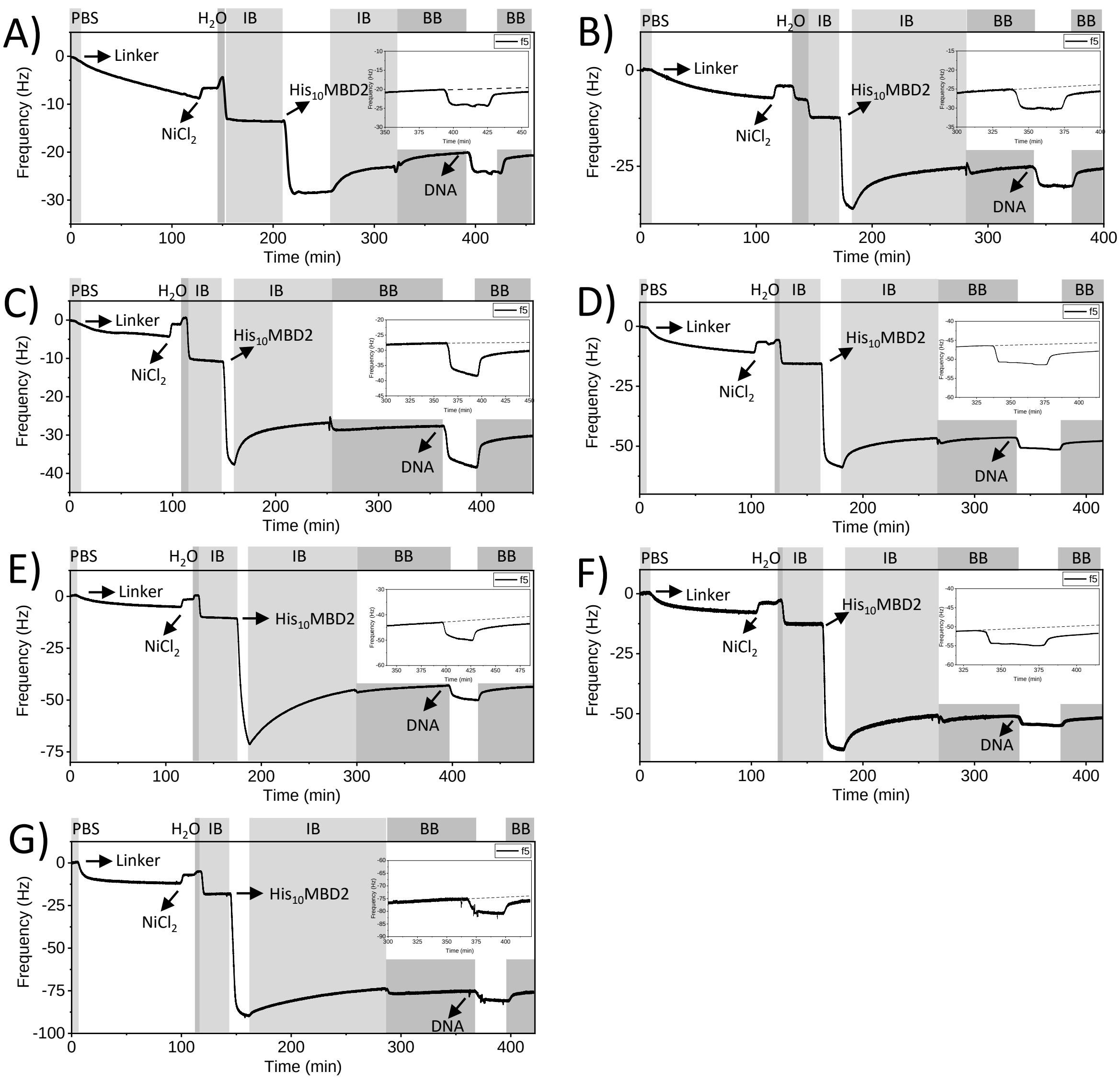


**Figure S12.** QCM time traces of the $His_{10}$MBD2 immobilization and subsequent DNA binding of Mal3C*pG. Prior to the $His_{10}$MBD2 immobilization, the SAM was formed, followed by linker binding and activation of the NTA groups by $NiCl_2$. The binding of Mal3C*pG far was monitored on MBD2 surface receptor densities of: A) $\Delta f$ = 10.5 Hz, B) $\Delta f$ = 12.3 Hz, C) $\Delta f$ = 14.5 Hz, D) $\Delta f$ = 30.8 Hz, E) $\Delta f$ = 31.0 Hz, F) $\Delta f$ = 36.9 Hz and G) $\Delta f$ = 54.1 Hz. Washing steps with PBS, Milli-Q ($H_2O$), IB and BB are indicated by the grey areas.

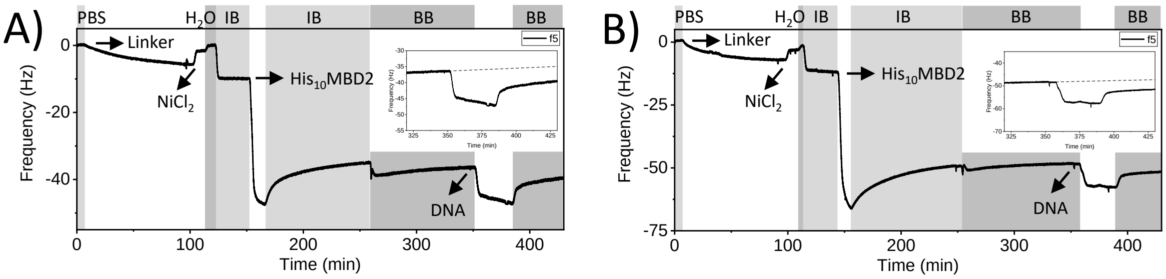


**Figure S13.** QCM times trace of the $His_{10}MBD2$ immobilization and subsequent DNA binding of Mal4C*pG. Prior to the $His_{10}MBD2$ immobilization, the SAM was formed, followed by linker binding and activation of the NTA groups by $NiCl_2$. The binding of Mal4C*pG far was monitored on MBD2 surface receptor densities of: A) $\Delta f$ = 23.6 Hz and B) $\Delta f$ = 32.7 Hz. Washing steps with PBS, Milli-Q ($H_2O$), IB and BB are indicated by the grey areas.

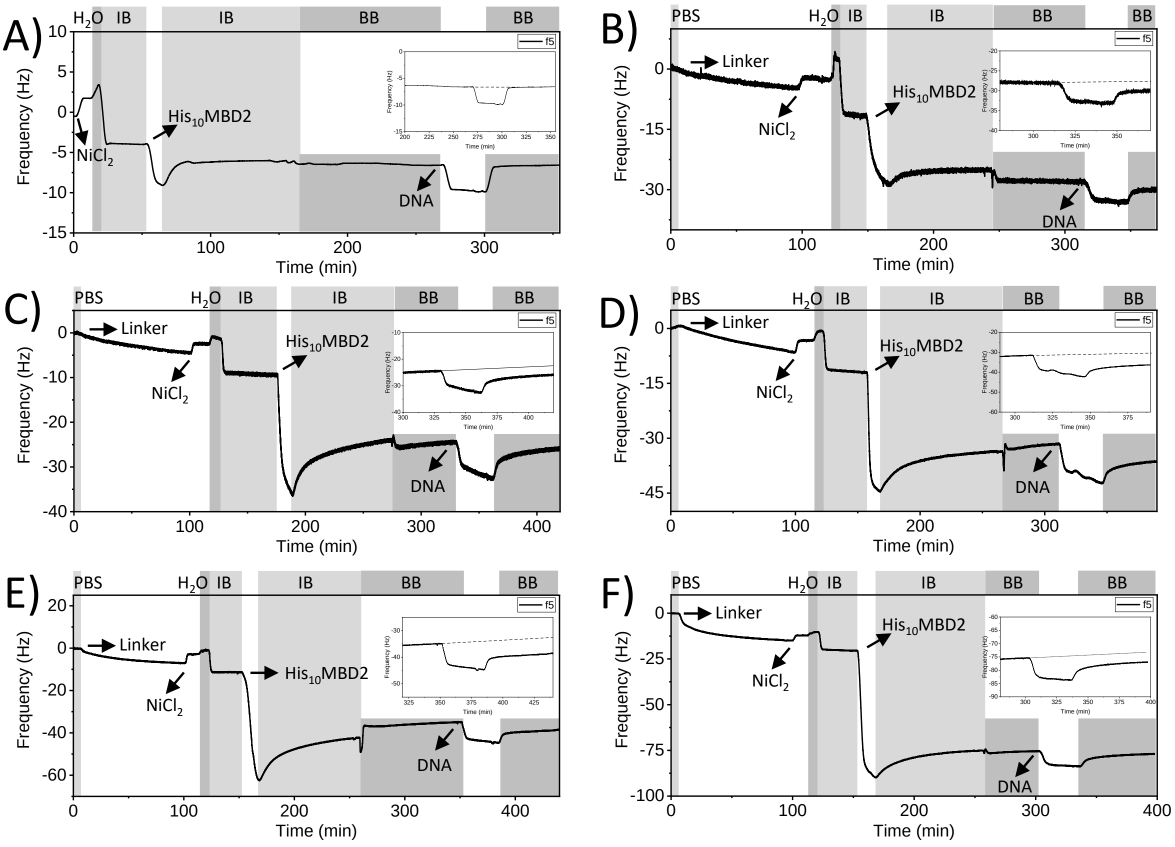


**Figure S14.** QCM times trace of the $His_{10}$MBD2 immobilization and subsequent DNA binding of Mal5C*pG. Prior to the $His_{10}$MBD2 immobilization, the SAM was formed, followed by linker binding and activation of the NTA groups by $NiCl_2$. The binding of Mal5C*pG was monitored on MBD2 surface receptor densities of: A) $\Delta f$ = 1.8 Hz, B) $\Delta f$ = 11.3 Hz, C) $\Delta f$ = 12.9 Hz, D) $\Delta f$ = 16.9 Hz, E) $\Delta f$ = 30.0 Hz and F) $\Delta f$ = 52.7 Hz. A) Does not displayer the linker molecule binding step. Washing steps with PBS, Milli-Q ($H_2O$), IB and BB are indicated by the grey areas.

## References

(1) Hendrich, B.; Bird, A. Identification and Characterization of a Family of Mammalian Methyl-CpG Binding Proteins. *Mol. Cell. Biol.* **1998**, *18* (11), 6538–6547. https://doi.org/10.1128/MCB.18.11.6538.
